# Non-Steroidal FXR Agonistic Dimeric 2-Methyl-4-(1-glycerol)furan with Lipid-Lowering Activities from Marine-Derived *Nocardiopsis* sp. ZSN1

**DOI:** 10.3390/md23030092

**Published:** 2025-02-20

**Authors:** Yongjun Jiang, Zhen Lei, Jiebin Fang, Yanping Wu, Chengpeng Sun

**Affiliations:** 1 School of Food and Pharmacy, Zhejiang Ocean University, Zhoushan 316021, China; 15274583055@163.com (Z.L.); wuyanping@zjou.edu.cn (Y.W.); 2 Institute of Marine Biology and Pharmacology, Ocean College, Zhejiang University, Zhoushan 316021, China; zyman@zju.edu.cn; 3 Tianjin State Key Laboratory of Therapeutic Substance of Traditional Chinese Medicine, School of Chinese Materia Medica, Tianjin University of Traditional Chinese Medicine, Tianjin 301617, China

**Keywords:** *Nocardiopsis* sp., 2-methyl-4-(1-glycerol)furan dimers, FXR agonistic activity, lipid-lowering activity

## Abstract

Five novel 2-methyl-4-(1-glycerol)furan (MGF) dimers, namely nocardifuran A (**1**), 13-*acetyl*-nocardifuran A (**2**), 15-*epi*-nocardifuran A (**3**), nocardifuran B (**4**), and nocardifuran C (**5**), were isolated from the Gause liquid fermentation of the marine-derived *Nocardiopsis* sp. ZSN1. Their structures were elucidated through HRESIMS, 1D and 2D NMR spectroscopic data analysis, and ECD calculations. Compounds **1**–**4** were identified as derivatives of MGF with its rearrangement of furan or pyran derivatives, while compound **5** was identified as the derivative of MGF with an indole derivative. These MGF dimers, representing a new structural class, were isolated from a marine microorganism for the first time, thereby enhancing chemical diversity. Screening for farnesoid X receptor (FXR) agonistic activity revealed that MGF dimers could activate FXR. Furthermore, bioactivity evaluations demonstrated that these types of compounds exhibited lipid-lowering activity with lower cytotoxicity in vitro. Consequently, our findings not only contribute to the chemical diversity of marine-derived MGF-type natural products but also offer potential insights into the development of MGF dimers as lead compounds for FXR agonists in the dysregulation of hepatic lipid metabolism.

## 1. Introduction

Due to the widespread dysregulation of hepatic lipid metabolism, non-alcoholic fatty liver disease (NAFLD) has emerged as the most prevalent liver disorder globally [1]. While patients diagnosed with NAFLD currently face limited therapeutic options, the promising potential of agonists targeting the nuclear receptor farnesoid X receptor (FXR) provides a beacon of hope. FXR, a member of the nuclear receptor superfamily with bile acids as its natural ligands, has been implicated in the occurrence and development of NAFLD according to various studies [2,3]. Activating FXR proves instrumental in regulating lipid metabolism within liver cells, controlling the release of inflammatory cytokines, alleviating liver inflammation, and impeding crucial processes in NAFLD, such as the formation of hepatic stellate cells and fibrosis. Numerous animal experiments and clinical studies have further substantiated the preventive and therapeutic effects of FXR agonists on NAFLD. Consequently, activating FXR emerges as a compelling and effective approach for both preventing and treating NAFLD [4,5,6].

Natural products and their derivatives play a significant role in drug development [7,8]. Several natural product molecules with FXR agonistic effects have been identified, including fargesone A [9], cafestol [10], coumestrol [11], and altenusin [12]. Despite an increasing number of clinical trials confirming the superiority of FXR agonists in treating NAFLD, the quantity of FXR agonists entering clinical research is limited, and many exhibit noticeable side effects. Hence, developing novel, safe, and effective FXR agonists is of paramount scientific significance.

**Figure 1:**
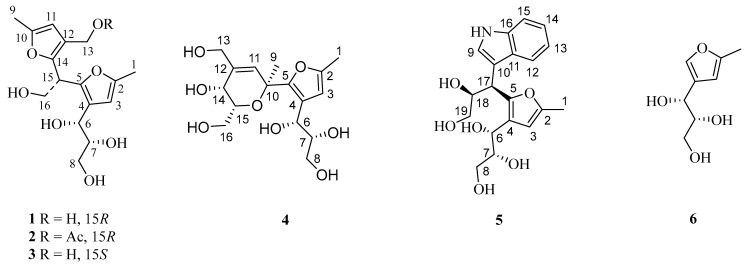
Chemical structures of compounds **1–6**.

In the course of our project focused on exploring chemical novelty and bioactive natural products from marine-derived actinomycetes, we selected a strain identified as *Nocardiopsis* sp. ZSN1 due to its crude extracts exhibiting in vitro lipid-lowering activities. A chemical investigation of *Nocardiopsis* sp. ZSN1 resulted in the identification of five new sphydrofuran-derived dimers (Figure 1), designated as nocardifuran A (**1**), 13-*acetyl*-nocardifuran A (**2**), 15-*epi*-nocardifuran A (**3**), nocardifuran B (**4**), and nocardifuran C (**5**), alongside a known analogue, 2-methyl-4-(1-glycerol)-furan (**6**) [13,14].

## 2. Results

The spores of the titled strain, cultivated on a Gause’s agar plate, were introduced into 500 mL Erlenmeyer flasks with 250 mL of Gause’s liquid medium. Subsequently, the flasks were incubated at 28 °C for 11 days on a rotary shaker (180 rpm). An ethyl acetate (EtOAc) extract obtained from the cultures (210 bottles) underwent separation through silica gel, Sephadex LH-20 column chromatography, and additional preparative HPLC purification, yielding six compounds, including five new dimeric 2-methyl-4-(1-glycerol)furan derivatives (**1**–**5**). The known compounds (**6**) were identified as 2-methyl-4-(1-glycerol)furan by comparing their spectroscopic data with literature-reported information [13,14].

Compound **1**, isolated as yellowish powder (methanol), was determined to have a molecular formula of C_16_H_22_O_7_ with six degrees of unsaturation, based on the analysis of its positive HRESIMS ion peak at *m/z* 349.1260 [M+Na]^+^. The careful examination of its ^1^H NMR data (Table 1 and Figure S1) revealed the presence of two aromatic protons at δ_H_ 5.97 (1H, s) and 5.97 (1H, s), as well as two methyl groups (δ_H_ 2.18 (3H, s) and 2.18 (3H, s)). Additionally, three oxygenated methylenes were observed [δ_H_ 3.09 (1H, m), 3.25 (1H, m), 3.80 (2H, dd, *J* = 5.5, 7.5 Hz), and 4.20 (2H, d, *J* = 5.5 Hz)], along with three methines, including two oxygenated ones at δ_H_ 3.37 (1H, m), 4.36 (1H, *J* = 6.5, 4.6 Hz), and 4.34 (1H, t, *J* = 7.6 Hz). The analysis of the ^13^C NMR data (Table 1 and Figure S2) of **1** revealed sixteen carbon signals, including two furan rings [δ_C_ 106.7, 107.5, 121.7, 122.7, 146.6, 146.6, 148.9, and 149.1]. Based on these findings and previous studies on the co-isolated antibiotic 2-methyl-4-(1-glycerol)furan (**6**) [13,14], compound **1** was identified as an analogue of the aforementioned antibiotic.

**Figure 2:**
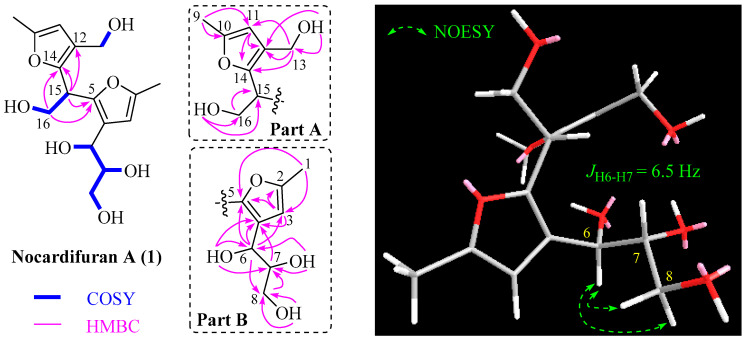
Selected 2D correlations for **1**.

By analyzing the HSQC, COSY, and HMBC correlations (Figure 2), the planar structure of compound **1** was determined. In the COSY spectrum, four spin systems were observed as follows: a, 6-OH/H-6/H-7/H-8/8-OH; b, 7-OH/H-7; c, 16-OH/H-16/H-15; d, 13-OH/H-13. The HMBC correlations of H-11 with C-10, C-12, and C-14 confirmed the trisubstituted furan ring in part A. The presence of the methyl group at C-10 and the hydroxy methylene group at C-12 was confirmed by HMBC cross-peaks between Me-9 and C-10 and C-11, and those between H_2_-13 and C-11, C-12, and C-14. The HMBC cross-peaks of H-15 with C-12 and C-14 and those of H-16 with C-14 confirmed the attachment of C-15 to C-14, establishing the structure of part A. Moving on to part B, the HMBC analysis confirmed the positions of Me-1 to C-2 due to the correlations of H-3 with C-2, C-4, and C-5, and of Me-1 with C-2 and C-3. The 6,7,8-trihydroxypropyl moiety is attached to C-4 of the furan ring due to the correlations of H-6 with C-3, C-4, and C-5, and of H-7 with C-4. Finally, the correlations between H-15 and H-16 with C-5 indicated that part A was connected to part B through the methine carbon (C-15). Thus, the planar structure of compound **1** was elucidated accordingly.

**Figure 3:**
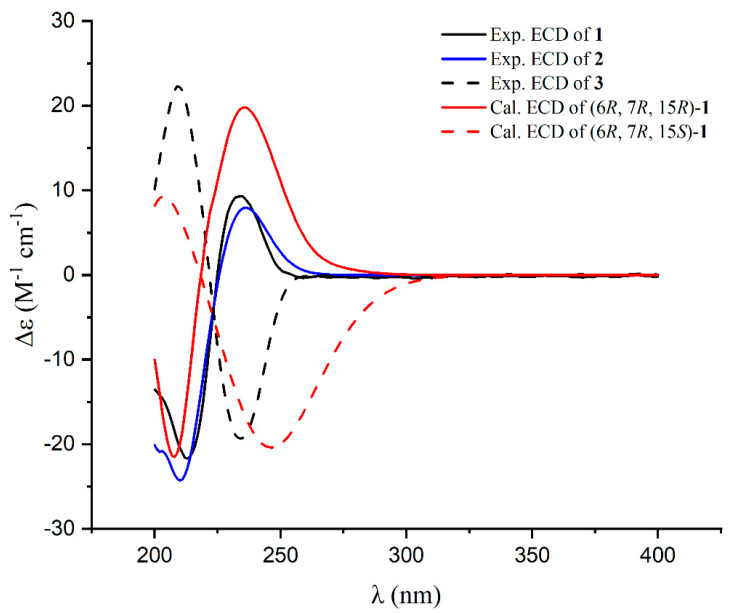
Experimental ECD spectra for **1–3** and calculated ECD spectra for **1**.

By referring to previous research, where the *R* configuration of C-6 and C-7 in the co-isolated 2-methyl-4-(1-glycerol)furan (**6**) was determined through chemical transformation and total synthesis [15,16,17], we propose that the fragment of 2-methyl-4-(1-glycerol)-furan in co-isolated compounds **1**–**5** also possesses an *R* configuration at C-6 and C-7 based on their shared same biosynthesis origin. However, the absolute configuration of the C-15 stereogenic carbon of **1** could not be established using NOESY and the coupling constant. To resolve this uncertainty, we employed the ECD method, conducting calculations for both possible isomers (6*R*, 7*R*, 15*R*; 6*R*, 7*R*, 15*S*). The ECD curve calculated for (6*R*, 7*R*, 15*R*)-**1** exhibited close conformity with the measured spectrum (Figure 3), confirming the absolute configuration as 6*R*, 7*R*, 15*R*. Consequently, the structure of **1** was conclusively identified as depicted and named nocardifuran A.

**Figure 4:**
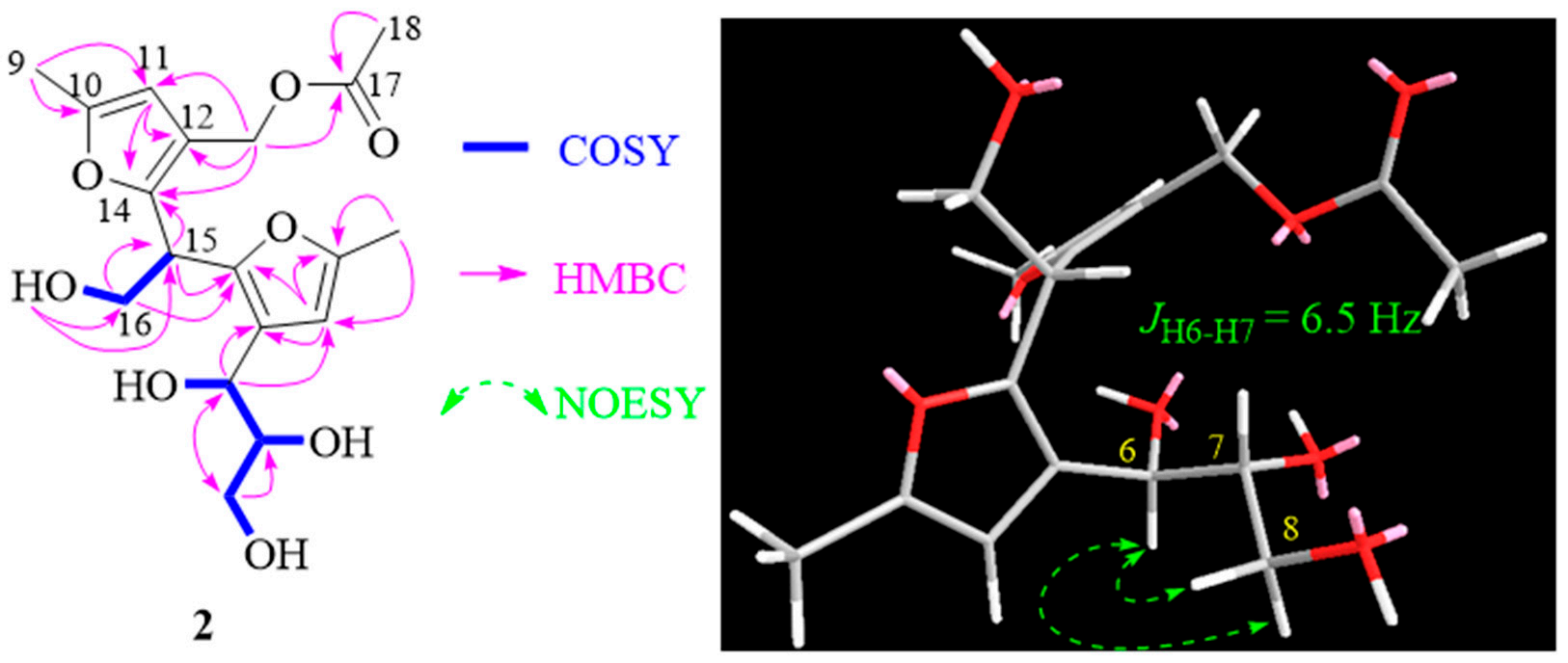
Selected 2D correlations for **2.**

Compound **2** exhibited a molecular formula of C_18_H_24_O_8_, deduced from its positive HRESIMS data. The ^1^H and ^13^C NMR spectra of **2** closely mirrored those of compound **1**, apart from an acetyl group at C-13 in **2** (Table 1). This observation aligns with the molecular formula and is further corroborated by HMBC correlations (see Figure 4). The congruent experimental ECD curves for compound **2** (Figure 3) collectively indicated that the absolute configuration is 6*R*, 7*R*, 15*R*, which is consistent with that of compound **1**. Consequently, the structure of **2** is confidently determined as illustrated and designated as 13-*acetyl*-nocardifuran A.

**Figure 5:**
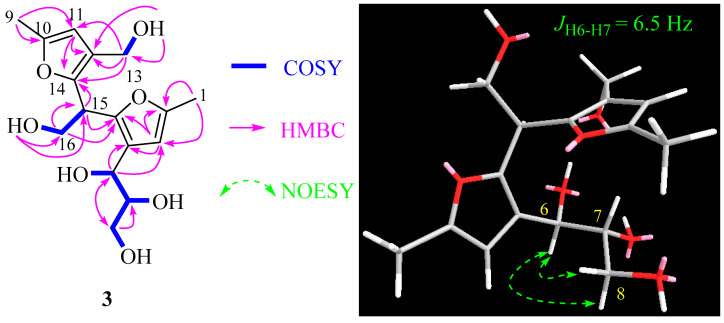
Selected 2D correlations for compound **3**.

Compound **3** exhibited an [M+Na]^+^ ion peak at *m/z* 349.1257 in its positive HRESIMS data, consistent with a molecular formula of C_16_H_22_O_7_. The ^13^C NMR data of **3** closely resembled those of **1** (Table 1). Small differences in the ^13^C NMR chemical shift for C-15 and C-16 in **3** compared to compound **1**, along with the data obtained from HMBC correlations (Figure 5) and the ECD spectrum (Figure 3), indicated a different stereochemistry at C-15. Consequently, the structure of **3** was unequivocally confirmed as 15-*epi*-nocardifuran A.

**Figure 6:**
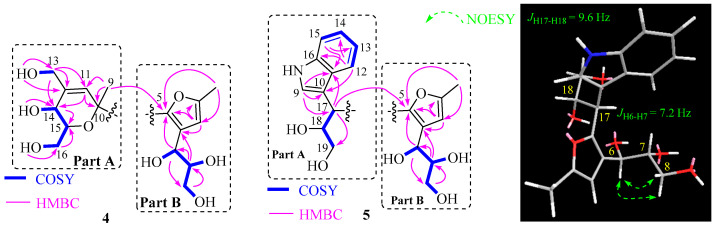
Selected 2D correlations for compounds **4** and **5**.

Nocardifuran B (**4**) was analyzed using HRESIMS, revealing a molecular formula of C_16_H_24_O_8_, indicating the presence of five degrees of unsaturation. The 1D NMR spectroscopic data (Table 2), combined with HSQC and HMBC correlations, showed that part B of compound **4** (Figure 6) was identical to that of co-isolated 2-methyl-4-(1-glycerol)furan. HMBC correlations confirmed the presence of the pyran ring in part A, specifically the correlations between H-11 and C-10, C-5, and C-14. The positions of Me-9 and the methylene hydroxyl groups at C-12 and C-15 were supported by HMBC correlations (Figure 6). A key HMBC correlation between Me-9 and C-5 indicated a linkage between part A and part B, elucidating the planar structure as shown.

**Figure 7:**
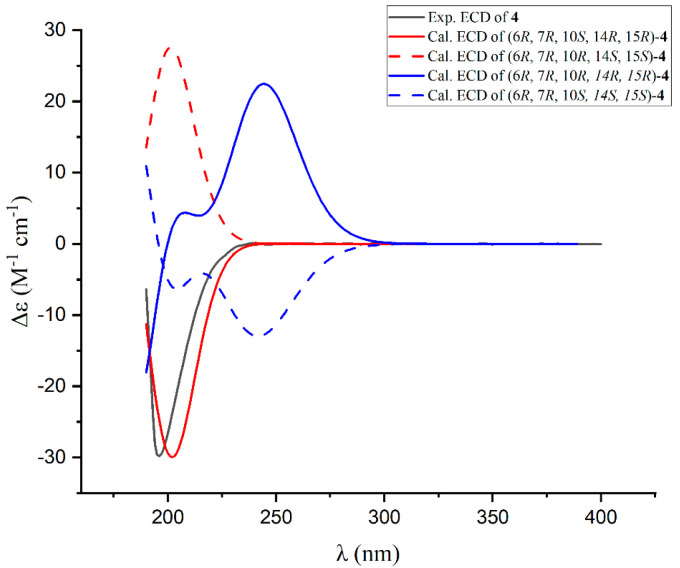
Experimental and calculated ECD spectra for compound **4**.

Additionally, the coupling constant between H-14 and H-15 (*J*_H-14/H-15_ = 2.0 Hz) suggests a *cis* configuration for these protons. To ascertain the absolute configurations of the pyran ring in compound **4**, ECD calculations were employed for the four potential isomers (6*R*, 7*R*, 10*S*, 14*R*, 15*R*; 6*R*, 7*R*, 10*R*, 14*S*, 15*S*; 6*R*, 7*R*, 10*R*, 14*R*, 15*R*; 6*R*, 7*R*, 10*S*, 14*S*, 15*S*). Notably, the calculated ECD curve of (6*R*, 7*R*, 10*S*, 14*R*, 15*R*)-**4** exhibited remarkable agreement with the experimental spectrum (Figure 7). This compelling correspondence unequivocally confirmed the absolute configuration of compound **4** as 6*R*, 7*R*, 10*S*, 14*R*, 15*R*, enhancing our understanding of its structural features.

Compound **5**, which was isolated as a yellow oily substance in methanol, displayed a molecular formula of C_19_H_23_NO_6_, indicating nine degrees of unsaturation. An analysis of the 1D NMR spectroscopic data, along with HSQC correlations, revealed the presence of a typical indole ring [δ_H_ 7.01 (1H, t, *J* = 7.8 Hz), 7.07 (1H, t, *J* = 7.8 Hz), 7.27 (1H, s), 7.31 (1H, d, *J* = 7.8 Hz), 7.71 (1H, d, *J* = 7.8 Hz)], a characteristic trisubstituted furan ring at δ_H_ 5.94 (1H, s), a methyl group at δ_H_ 2.27 (3H, s), two oxygenated methylene groups at δ_H_ 3.58 (1H, dd, *J* = 12.0, 3.0 Hz), 3.50 (1H, dd, *J* = 11.4, 3.0 Hz), and 3.35 (1H, m), as well as four methines including three oxygenated carbons [δ_H_ 3.70 (1H, m), 4.74 (1H, d, *J* = 7.2 Hz), and 4.34 (1H, m)].

The 2D NMR correlations as shown in Figure 6 confirmed that part B of compound **5** was identical to the 2-methyl-4-(1-glycerol)furan. The indole ring of part A was confirmed through HMBC correlations. The attachment of the -CHCHOHCH_2_OH fragment to C-10 was supported by the ^1^H-^1^H COSY correlations of H-17/H-18/H19, as well as HMBC correlations between H-17 and C-9, C-10, C-18, and C-19. Finally, the key HMBC correlation between H-17 and C-5 confirmed the connection between part A to part B.

**Figure 8:**
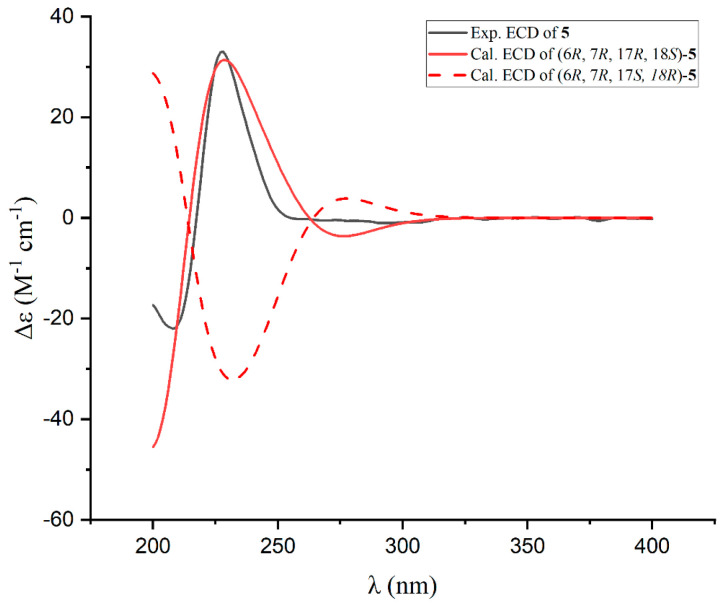
Experimental and calculated ECD spectra for compound **5**.

The coupling constant of H-17/H-18 (*J*_H-17/H-18_ = 9.6 Hz) indicated a *threo* configuration for these protons. To determine the absolute configurations of C-17 and C-18, ECD calculations were performed for two possible diastereomers: (6*R*, 7*R*, 17*R*, 18*S*)-**5** and (6*R*, 7*R*, 17*S*, 18*R*)-**5**. The calculated ECD spectrum of (6*R*, 7*R*, 17*R*, 18*S*)-**5** closely matched the experimental data (Figure 8), unambiguously establishing the 6*R*, 7*R*, 17*R*, 18*S* configuration. This confirms compound 5 as nocardifuran C.

**Scheme 1:**
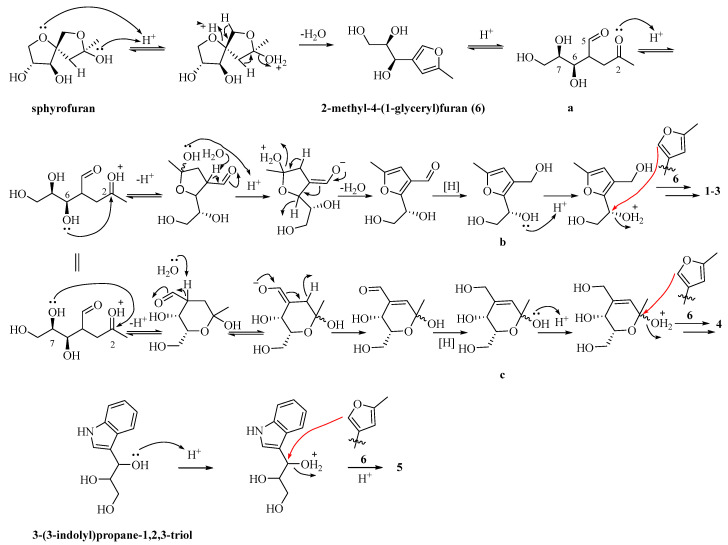
Proposed biosynthetic pathways for compounds **1–6**.

In biosynthesis, the genesis of novel compounds **1**–**5** and the established compound **6** can be retraced to sphydrofuran, characterized by anomeric and ring–chain tautomeric isomerism (Scheme 1). The conversion of sphydrofuran into 2-methyl-4-(1-glycerol)furan (**6**) is feasibly accomplished through a sequential process involving dehydration and subsequent ring-opening elimination. Under acidic conditions, compound **6** experiences furan ring-opening, resulting in the generation of the 2,5-dione intermediate **a**. The 6-OH or 7-OH groups of intermediate **a**, in conjunction with the 2-carbonyl group, undergo intramolecular nucleophilic addition, facilitating subsequent dehydration and reduction. This process ultimately yields either the furan derivative **b** or the pyran ring derivative **c**. In the context of the furan derivative **b**, under acidic conditions, the protonation of the hydroxyl group at the 7-OH position initiates an electrophilic substitution reaction at the C-5 position of compound **6**, leading to the formation of dimeric **1**–**3**. Similarly, in the case of the pyran derivative **c**, under acidic conditions, the protonation of the 6-OH group occurs, causing its involvement in an electrophilic substitution reaction at the C-5 position of compound **6**, resulting in the formation of dimeric **4**. Compound **5** is produced through an electrophilic substitution reaction between 3-(3-indolyl)propane-1,2,3-triol and compound **6** under acidic conditions.

**Figure 9:**
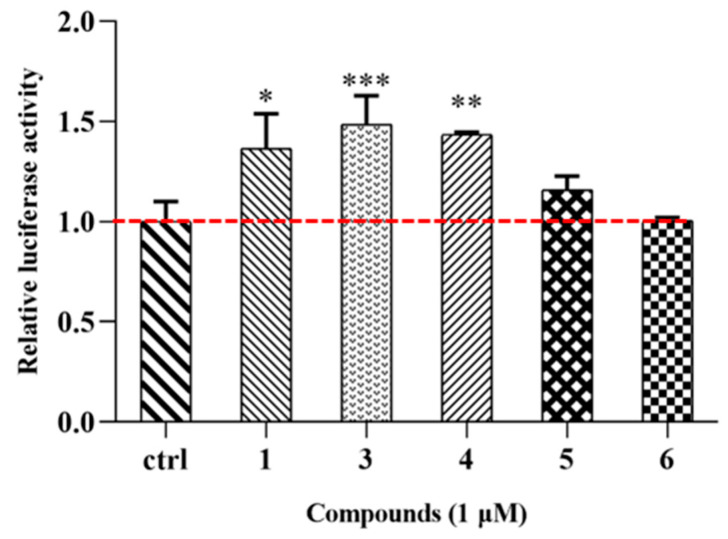
Compounds **1** and **3**−**6** (1 μM) activate FXR transactivation activity. * *p* < 0.05 versus control group, ** *p* < 0.01 versus control group, *** *p* < 0.001 versus control group (ctrl refers to the control group).

Lipid metabolism disorder encompasses disruptions in fat synthesis, breakdown, transport, and storage, leading to abnormal lipid levels. FXR, part of the nuclear receptor superfamily with bile acids as natural ligands, can activate and regulate genes associated with lipid metabolism [18]. Therefore, FXR is a crucial target for treating lipid metabolism disorders. The study’s outcomes reveal that novel MGF dimers **1**, **3**–**4** exhibit significant agonistic effects on FXR at a concentration of 1 μM (Figure 9). These findings suggest that MGF dimers have the potential to be promising candidates for further development as new FXR agonists.

**Figure 10:**
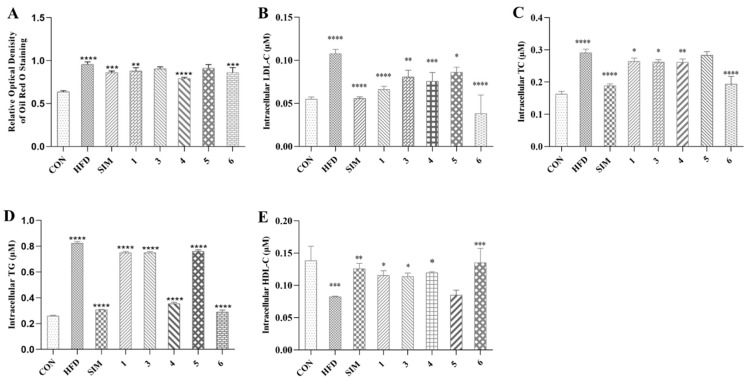
(**A**) Quantification analysis by Oil Red O staining. Intracellular LDL-C (**B**), TC (**C**), TG (**D**), and HDL-C (**E**). Data were the means ± standard deviation (SD) calculated by Prism, and each group was compared with HFD group; * *p* < 0.05, ** *p* < 0.01, *** *p* < 0.001, **** *p* < 0.0001 (one-way ANOVA). CON: Control group, which refers to the baseline or untreated group used for comparison in the experiment. HFD: High-fat diet, referring to the group fed a high-fat diet to induce conditions such as obesity and metabolic disorders. SIM: Simvastatin, a drug commonly used in research to evaluate its effects on lipid metabolism and related conditions.

The MTT assay results demonstrated that at a concentration of 100 μM, all tested compounds had no discernible impact on HepG2 cell line growth, affirming their biological safety for lipid-lowering applications. To assess the compounds’ influence on in vitro lipid accumulation reduction, we employed a lipid-loaded model of HepG2 cells exposed to 0.45 mM fatty acids (oleic acid: palmitic acid = 2:1) for 24 h. A quantitative analysis revealed that compounds **1** and **3**–**4**, at concentrations of 10 μM, effectively reduced intracellular fat deposition, comparable to the positive control simvastatin (Figure 10A). Additionally, we assessed intracellular LDL-C, TG, TC, and HDL-C levels in HepG2 cells. Figure 10B–E demonstrate that fatty acid treatment significantly increased LDL-C, TG, and TC levels. MGF dimers **1** and **3**–**4**, at a concentration of 10 μM, markedly decreased intracellular LDL-C, TC, and TG levels (Figure 10B–D) while elevating intracellular HDL-C levels (Figure 10E). These findings suggest that MGF dimers effectively alleviate lipid accumulation in HepG2 cells induced by fatty acids.

## 3. Discussion

The discovery of five novel MGF dimers (**1**–**5**) from the marine-derived *Nocardiopsis* sp. ZSN1 represents a significant advancement in the field of marine natural product chemistry. These compounds, characterized as dimeric derivatives of 2-methyl-4-(1-glycerol)furan (MGF), exhibit unique structural features, including rearranged furan/pyran rings (compounds **1**–**4**) and an unprecedented indole-containing derivative (compound **5**). Their isolation from a marine microorganism marks the first report of such structures in this ecological niche, expanding the chemical diversity of marine-derived secondary metabolites.

The agonistic activity of compounds **1**, **3**, and **4** against FXR at 1 μM underscores their therapeutic potential in addressing lipid metabolism disorders, particularly NAFLD. FXR activation has been extensively validated as a key strategy for regulating hepatic lipid homeostasis, suppressing inflammation, and mitigating fibrosis in NAFLD models [2,3,4]. The observed FXR transactivation activity of these MGF dimers aligns with the pharmacological profiles of other natural FXR agonists, such as fargesone A and altenusin [9,12]. However, the distinct structural scaffold of MGF dimers may offer advantages in terms of selectivity and reduced off-target effects compared to existing agonists, which often face challenges like hepatotoxicity or limited efficacy in clinical trials [6]. Notably, the low cytotoxicity of these compounds (up to 100 μM in HepG2 cells) further enhances their candidacy for further drug development, addressing a critical bottleneck in the translation of FXR-targeted therapies.

The lipid-lowering efficacy of compounds **1**, **3**, and **4** in reducing intracellular lipid accumulation (LDL-C, TG, TC) and elevating HDL-C levels in HepG2 cells highlights their dual role as FXR activators and metabolic modulators. These effects mirror those of simvastatin, a clinically used lipid-lowering agent, yet the MGF dimers operate through a distinct mechanism by directly targeting nuclear receptors. This dual functionality—combining receptor activation and metabolic regulation—positions these compounds as promising leads for multi-target therapies in metabolic syndrome-associated conditions. The structural diversity within the MGF dimer series (e.g., acetylated or epimerized derivatives) also provides a foundation for structure–activity relationship (SAR) studies to optimize potency and pharmacokinetic properties. However, the current study is limited to in vitro evaluations; future investigations should prioritize in vivo models to validate their efficacy and safety in a physiological context. Additionally, exploring the pharmacokinetic profiles, tissue distribution, and long-term toxicity of these compounds will be critical for advancing them toward preclinical development.

In conclusion, this study not only enriches the repertoire of marine-derived FXR agonists but also underscores the untapped potential of marine actinomycetes in drug discovery. The structural novelty, bioactivity, and low cytotoxicity of MGF dimers position them as compelling candidates for further exploration as therapeutics for NAFLD and related metabolic disorders. Future efforts should focus on mechanistic studies to elucidate their interactions with FXR and other metabolic targets, as well as translational research to bridge the gap between bench discoveries and clinical applications.

## 4. Materials and Methods

### 4.1. General Experimental Procedures

The 600 MHz JEOL JNM-ECZR instrument was utilized for acquiring 1D and 2D NMR spectra. Specifically, ^1^H NMR spectra were recorded at 600 MHz, and ^13^C NMR spectra were recorded at 150 MHz. TMS served as the internal standard. Positive HR-ESI-MS data were evaluated using an Agilent 1260 HPLC-6230 TOF tandem spectrometer (Agilent Technologies, Santa Clara, CA, USA). IR and UV analyses were performed using the BURKER TENSOR II FT-IR spectrometer platform (Bruker Corporation, Billerica, MA, USA) and a SHIMAZU UV-1800 ultraviolet spectrophotometer (Shimadzu Corporation, Kyoto, Japan), respectively. Optical rotations were determined through AUTOPOL I digital polarimetry (Rudolph Research Analytical, Hackettstown, NJ, USA), and the ECD spectrum was recorded using the JASCO J-1700 spectrometer (JASCO Corporation, Tokyo, Japan). Thin-layer chromatography (TLC) utilized precoated silica gel GF254 plates from Qingdao Haiyang Chemical Co., Ltd., Qingdao, China. Column chromatography (CC) was conducted on silica gel (300–400 meshes or 100–200 meshes, Qingdao Haiyang Chemical Company, Qingdao, China) and Sephadex LH-20 (Amersham Pharmacia Biotech, Uppsala, Sweden). Preparative HPLC was carried out on a Beijing auno tech LC-2000 system equipped with an Agilent C18 column (10 μm, 21.2 × 250 mm). All solvents used for CC were of analytical grade (Shanghai Chemical Reagents Co., Ltd., Shanghai, China), and HPLC-grade solvents (J & K Scientific Ltd., Beijing, China) were employed for HPLC.

HEK293T and HepG2 cells were obtained from the Cell Bank of the Chinese Academy of Sciences in Shanghai, China. Fetal bovine serum (FBS), Minimum Essential Medium (MEM), phosphate-buffered saline (PBS), trypsin–EDTA (0.25%), penicillin–streptomycin, alanine acid, L-glutamic acid, and MEM non-essential amino acids were acquired from Gibco (Thermo Scientific, Waltham, MA, USA). Sodium oleate and sodium palmitate, simvastatin, and Oil Red O were obtained from Sigma (St. Louis, MO, USA). The 4% PFA Fix Solution was purchased from Beyotime Biotechnology Institute. The TG assay kit, TC assay kit, and LDL-C assay kit were acquired from Nanjing Jiancheng Bioengineering Institute (Nanjing, China).

### 4.2. Strain Isolation and Identification

The ZSN1 strain was isolated from marine sediments located at Zhoushan Island, Zhejiang Province, China, using the standard dilution plating method. BGI Genomics Co., Ltd. (Shenzhen, China) conducted the strain identification through 16S rDNA sequence analysis. The obtained DNA sequence underwent a BLAST search (nucleotide sequence comparison) against the GenBank database. The results of the BLAST analysis revealed a 99.68% similarity between the strain’s sequence and that of *Nocardiopsis* sp. AD16 (accession number MF410670.1), confirming its classification within the genus *Nocardiopsis*. The sequence of the strain has been deposited in GenBank under accession number MN736491. Currently, the strain is preserved at the Laboratory of the School of Food and Pharmacy, Zhejiang Ocean University.

### 4.3. Fermentation, Extraction, Isolation, and Purification

Colonies of the ZSN1 strain, cultivated on Gause’s agar plates, were transferred to a 500 mL Erlenmeyer flask containing 200 mL of Gause’s liquid medium. The inoculated flask was then incubated at 28 °C for 11 days on a rotary shaker (180 rpm) to facilitate fermentation. A total of 210 bottles were meticulously prepared for subsequent investigations. The entire fermented culture underwent triple extraction with ethyl acetate at room temperature. The resultant ethyl acetate fraction was subjected to vacuum drying, yielding a crude EtOAc extract (4.6 g), which was subsequently processed through MPLC (ODS, 200 g) with elution using methanol–water gradients (ranging from 1:9 to 1:0). This process produced seven distinct fractions (A–G).

In the initial step, Fraction D (123 mg) was separated using Sephadex LH-20, with methanol as the eluting solvent, resulting in the formation of three sub-fractions (D1-3). Subsequently, sub-fraction D-3 (26 mg) underwent purification through preparative HPLC (45–60% methanol in water, flow rate 10 mL/min), culminating in the isolation of Compound **5** (3.2 mg, t_R_ = 13.5 min).

Fraction E (563 mg) was initially separated using Sephadex LH-20 and methanol as the eluting solvent, resulting in two sub-fractions (E1–2). Further purification of sub-fraction E-1 (110 mg) was accomplished through preparative HPLC (10–100% methanol in water, flow rate 10 mL/min), leading to the isolation of compounds **1** (4.8 mg, t_R_ = 14.0 min), **2** (5.6 mg, t_R_ = 16.0 min), and **3** (3.2 mg, t_R_ = 20 min). Compound **6** (12 mg) was isolated from sub-fraction E-2 (46 mg) using silica gel and elution with a solvent mixture of dichloromethane with methanol (25:1).

Finally, fraction F (67 mg) was purified using preparative HPLC (10–100% methanol in water, flow rate 10 mL/min), resulting in the yield of compound **4** (7.6 mg, t_R_ = 7.5 min).

### 4.4. Characterization Data

#### 4.4.1. Nocardifuran A (**1**)

Yellowish powder (methanol); [α]^20^_D_ -24 (*c* 0.1, methanol); UV (methanol) λ_max_ (log ε): 219 (3.92), 229 (3.94) nm; IR ν_max_: 3419, 1681, 1203, 1137 cm^−1^; ^1^H and ^13^C NMR data, see Table 1; HRESIMS *m*/*z* 349.1260 [M+Na]^+^ (calcd for C_16_H_22_NaO_7_, 349.1263).

#### 4.4.2. 13-Acetyl-nocardifuran A (**2**)

Yellowish powder (methanol); [α]^20^_D_ -22 (*c* 0.1, methanol); UV (methanol) λ_max_ (log ε): 219 (3.85), 229 (3.87) nm; IR ν_max_: 3421, 1639, 1201, 1103 cm^−1^; ^1^H and ^13^C NMR data, see Table 1; HRESIMS *m*/*z* 391.1365 [M+Na]^+^ (calcd for C_18_H_2_4NaO_8_, 391.1369).

#### 4.4.3. 15-Epi-nocardifuran A (**3**)

Yellowish powder (methanol); [α]^20^_D_ -13.2 (*c* 0.1, methanol); UV (methanol) λ_max_ (log ε): 219 (3.92), 229 (3.94) nm; ^1^H and ^13^C NMR data, see Table 1; HRESIMS *m*/*z* 349.1260 [M+Na]^+^ (calcd for C_16_H_22_NaO_7_, 349.1263).

#### 4.4.4. Nocardifuran B (**4**)

Yellowish powder (methanol); [[α]^20^_D_ 32.0 (*c* 0.1, methanol); UV (methanol) λ_max_ (log ε): 223 (3.82) nm; ^1^H and ^13^C NMR data, see Table 2; HRESIMS *m*/*z* 367.1367 [M+Na]^+^ (calcd for C_16_H_24_NaO_8_, 367.1365).

#### 4.4.5. Nocardifuran C (**5**)

Yellowish oil (methanol); [α]^20^_D_ -6.7 (c 0.1, methanol); UV (methanol) λ_max_ (log ε): 223 (3.75), 276 (2.78) nm; ^1^H and ^13^C NMR data, see Table 2; HRESIMS *m*/*z* 384.1416 [M+Na]^+^ (calcd for C_19_H_23_NNaO_6_, 384.1423).

#### 4.4.6. 2-Methyl-4-(1-glycerol)-furan (**6**)

Yellowish oil (methanol); ^1^H and ^13^C NMR, and HRESIMS data, see in the reference [17].

### 4.5. ECD and NMR Calculations

The conformational analysis of compounds **1**–**2**, **4,** and **5** utilized the xtb software package (version 3) for an initial search, followed by DFT/TDDFT calculations using Gaussian 16 with the GNF0-xTB semiempirical method for optimization [19]. Conformations with energy differences below 0.5 kcal/mol and geometric distances below 0.5 angstroms were merged, and frequency calculations were performed to estimate relative thermal free energies (ΔG) at 298.15 K. Low-energy conformers in methanol were computed at the B3LYP/def2-QZVPP level considering solvent effects via the polarizable continuum model (PCM).

NMR calculations at the B3LYP/def2TZVP level determined chemical shifts using the GIAO method, establishing relative configurations through DP4+ probability calculations based on Boltzmann-weighted distributions at 298.15 K. ECD calculations employed the PBE0 hybrid functional and Ahlrichs’ def2-TZVP basis sets, generating spectra with Gaussian View 5.0 software. Spectra were adjusted based on the equilibrium population and Boltzmann weighting of each conformer in a methanol solution. The calculated ECD spectra were further adjusted for discrepancies with experimental UV absorption peaks [20].

### 4.6. Agonistic Activities of Isolated Compounds Against FXR

The expression vector containing hFXR and the BSEP promoter luciferase reporter was utilized. Agonistic effects against FXR were assessed for all compounds, except for compound **3**, following previously established procedures [18]. HEK293T cells were transfected with FXRα2 expression plasmids along with 25 ng of an internal reference. Following the addition of reagents, cells were incubated for 11 h, and subsequently harvested in luciferase lysis buffer for the detection of luciferase activity (Promega Corporation, Madison, USA). The obtained luciferase activity was normalized with renilla luciferase activity.

### 4.7. Cytotoxicity Assay and Lipid-Lowering Activity Assay

HepG2 cells were cultured in Minimum Essential Medium supplemented with 10% FBS, 1% Penicillin–streptomycin, 1% Alanine acid, and 1% L-glutamic acid at 37 °C in a 5% CO_2_ incubator. Cell digestion with trypsin–EDTA (0.25%) occurred every 3 days, and cell passage was performed at a 1:2 ratio, selecting cells in the logarithmic stage for experiments.

Cytotoxicity was assessed through the MTT assay. HepG2 cells were seeded in 96-well culture plates at approximately 10,000 cells per well and incubated at 37 °C in a 5% CO_2_ incubator for 24 h. Control and tested compound groups were established with final concentrations, each with 4 parallel wells. After 24 h of culture, 20 µL of 0.5 mg/mL MTT solution was added to each well and incubated for an additional 4 h. The medium was then removed, and 150 µL of DMSO was added to each well. The optical density (OD) at 490 nm was measured using an enzyme marker. The cell survival rate was calculated as follows: OD tested compound group 490 nm/OD control group 490 nm × 100%.

HepG2 cells were seeded in 12-well culture plates with approximately 300,000 cells per well and incubated at 37 °C in a 5% CO_2_ incubator for 24 h. The control group, model group, positive control group, and tested compounds groups were established, each with 4 parallel wells. The control group received 1 mL of blank MEM, the model group received a mixture of sodium oleate (0.6 mM) and sodium palmitate (0.3 mM) (1 mL), and the tested compounds group received 0.5 mL of the lipid mixture and 0.5 mL of the corresponding compound concentration solution. After 24 h, each well was rinsed three times with PBS, fixed with 4% PFA Fix Solution for 40 min, washed with PBS three times, and stained with Oil Red O working solution for 40 min.

## Figures and Tables

**Table 1:** ^1^H NMR (600 MHz) and ^13^C NMR (150 MHz) spectroscopic data of compounds **1–3.**

	1 ^a^		2 ^a^		3 ^a^	
no.	δ_H_, *J* in Hz	δ_C_, Type	δ_H_, *J* in Hz	δ_C_, Type	δ_H_, *J* in Hz	δ_C_, Type
1	2.18, s	13.4, CH_3_	2.18, s	13.4, CH_3_	2.18, s	13.3, CH_3_
2		149.1, C		149.2, C		149.0, C
3	5.97, s	106.7, CH	5.97, s	106.7, CH	5.97, s	106.3, CH
4		122.7, C		122.9, C		122.4, C
5		146.6, C		146.1, C		146.5, C
6	4.36, dd (6.5, 4.6)	65.6, CH	4.34, dd (6.5, 4.6)	65.7, CH	4.40, dd (6.5, 4.6)	65.8, CH
7	3.37, m	75.2, CH	3.37, m	75.3, CH	3.45, m	75.2, CH
8	3.25, m	62.5, CH_2_	3.21, m	62.5, CH_2_	3.36, m	62.5, CH2
	3.09, m		3.05, m		3.17, m	
9	2.18, s	13.4, CH_3_	2.19, s	13.3, CH_3_	2.19, s	13.3, CH_3_
10		148.9, C		149.9, C		149.0, C
11	5.96, s	107.5, CH	5.98, s	107.8, CH	5.97, s	107.5, CH
12		121.7, C		116.2, C		121.6, C
13	4.20, d (5.5)	54.4, CH_2_	4.83, d (12.5)	57.3, CH_2_	4.21, dd (5.5, 3.0)	54.3, CH_2_
			4.76, d (12.5)			
14		146.6, C		149.4, C		146.5, C
15	4.34, t (7.6)	38.3, CH	4.40, t (7.6)	38.3, CH	4.33, t (7.8)	37.8, CH
16	3.80, dd (7.6, 5.5)	61.9, CH_2_	3.79, dd (7.6, 5.5)	61.9, CH_2_	3.82, dd (7.8, 5.4)	61.5, CH_2_
17				170.4, C		
18			1.99, s	20.7, CH_3_		
19						
6-OH	4.69, d (4.6)		4.71, d (6.0)		4.72, d (4.6)	
7-OH	4.55, d (5.2)		4.57, d (5.2)		4.60, d (4.6)	
8-OH	4.41, t (5.5)		4.34, t (5.5)		4.38, t (5.5)	
13-OH	4.70, t (5.5)				4.69, t (5.5)	
14-OH						
16-OH	4.81, t (5.5)		4.84, t (5.5)		4.82, t (5.5)	

^a^ Recorded in DMSO-*d_6_.*

**Table 2:** ^1^H NMR (600 MHz) and ^13^C NMR (150 MHz) spectroscopic data of compounds **4–5.**

	4 ^a^		5 ^b^	
no.	δ_H_, *J* in Hz	δ_C_, Type	δ_H_, *J* in Hz	δ_C_, Type
1	2.17, s	13.3, CH_3_	2.27, s	13.6, CH_3_
2		148.6, C		151.6, C
3	6.07, s	107.6, CH	5.94, s	106.2, CH
4		124.7, C		122.0, C
5		147.8, C		151.7, C
6	4.73, dd (6.5, 4.8)	64.1, CH	4.74, d (7.2)	68.7, CH
7	3.42, m	75.1, CH	3.70, m	77.3, CH
8	3.37, m	62.7, CH_2_	3.58, dd (12.0, 3.0)	64.3, CH_2_
	3.21, m		3.35, m	
9	1.46, s	28.4, CH_3_	7.27, s	124.1, CH
10		73.3, C		114.4, C
11	5.87, s	126.1, CH		128.1, C
12		138.3, C	7.71, d (7.8)	120.0, CH
13	4.0, d (5.0)	61.7, CH_2_	7.01, t (7.8)	119.9, CH
14	3.67, dd (7.2, 2.0)	60.4, CH	7.07, t (7.8)	122.5, CH
15	3.35, m	74.1, CH	7.31, d (7.8)	112.3, CH
16	3.55, m	60.1, CH_2_		120.0, C
17			4.49, d (9.6)	38.6, CH
18			4.34, m	75.7, CH
19			3.50, dd (11.4, 3.0)	66.0, CH_2_
6-OH	4.52, d (6.0)			
7-OH	4.48, d (5.5)			
8-OH	4.59, t (5.5)			
13-OH	4.76, t (5.0)			
14-OH	4.45, d (7.2)			
16-OH	4.44, t (2.5)			

**^a, b^** Recorded in DMSO-*d_6_* and methanol-*d_4_*, respectively.

## Data Availability

The original data presented in the study are included in the article/Supplementary Materials; further inquiries can be directed to the corresponding author.

## Supplementary Materials

The following supporting information can be downloaded at: https://www.mdpi.com/article/10.3390/md23030092/s1, Figures S1–S41:1D (^1^H and ^13^C), and 2D (COSY, HSQC, HMBC, NOESY) NMR, HRESIMS, UV and IR spectra of compounds **1**–**5**.
