# Supplementary material for: Non-Steroidal FXR Agonistic Dimeric 2-Methyl-4-(1-glycerol)furan with Lipid-Lowering Activities from Marine-Derived Nocardiopsis sp. ZSN1
Source: Mar Drugs. 2025 Feb 20;23(3):92. doi: 10.3390/md23030092 (PMC11944181; doi:10.3390/md23030092)
Supplement: Supplementary file 1 [file marinedrugs-23-00092-s001.zip › marinedrugs-3467568-supplementary.pdf]

Supplementary Information

Non-steroidal FXR agonists dimeric 2-methyl-4-(1-glycerol)furan with lipid-lowering activities from marine-derived *Nocardiopsis* sp. ZSN1

Yongjun Jiang [1,*], Zhen Lei [1], Jiebin Fang [3], Yanping Wu [1], Chengpeng Sun [2,*]

[1] School of Food and Pharmacy, Zhejiang Ocean University, Zhoushan 316021, People's Republic of China;

[2] School of Chinese Materia Medica, Tianjin State Key Laboratory of Therapeutic Substance of Traditional Chinese Medicine, Tianjin University of Traditional Chinese Medicine, Tianjin 301617, People's Republic of China;

[3] Institute of Marine Biology and Pharmacology, Ocean College, Zhejiang University, Zhoushan 316021, People's Republic of China

* Correspondence: jiangyj@zjou.edu.cn, suncp146@163.com

**Contents**	Pages
**Figures S1-S6.** 1D and 2D NMR of **1**	3-5
**Figure S7** HRESIMS spectrum of **1**	6
**Figure S8** IR spectrum of **1**	6
**Figure S9** UV spectrum of **1**	6
**Figures S10-S15.** 1D and 2D NMR of **2**	7-9
**Figure S16** HRESIMS spectrum of **2**	10
**Figure S17** IR spectrum of **2**	10
**Figure S18** UV spectrum of **2**	11
**Figures S19-S24.** 1D and 2D NMR of **3**	11-13
**Figure S25** HRESIMS spectrum of **3**	14
**Figure S26** UV spectrum of **3**	14
**Figures S27-S32.** 1D and 2D NMR of **4**	15-17
**Figure S33** HRESIMS spectrum of **4**	18
**Figure S34** UV spectrum of **4**	18
**Figures S35-S40** 1D and 2D NMR of **5**	19-21
**Figure S41** HRESIMS spectrum of **5**	22

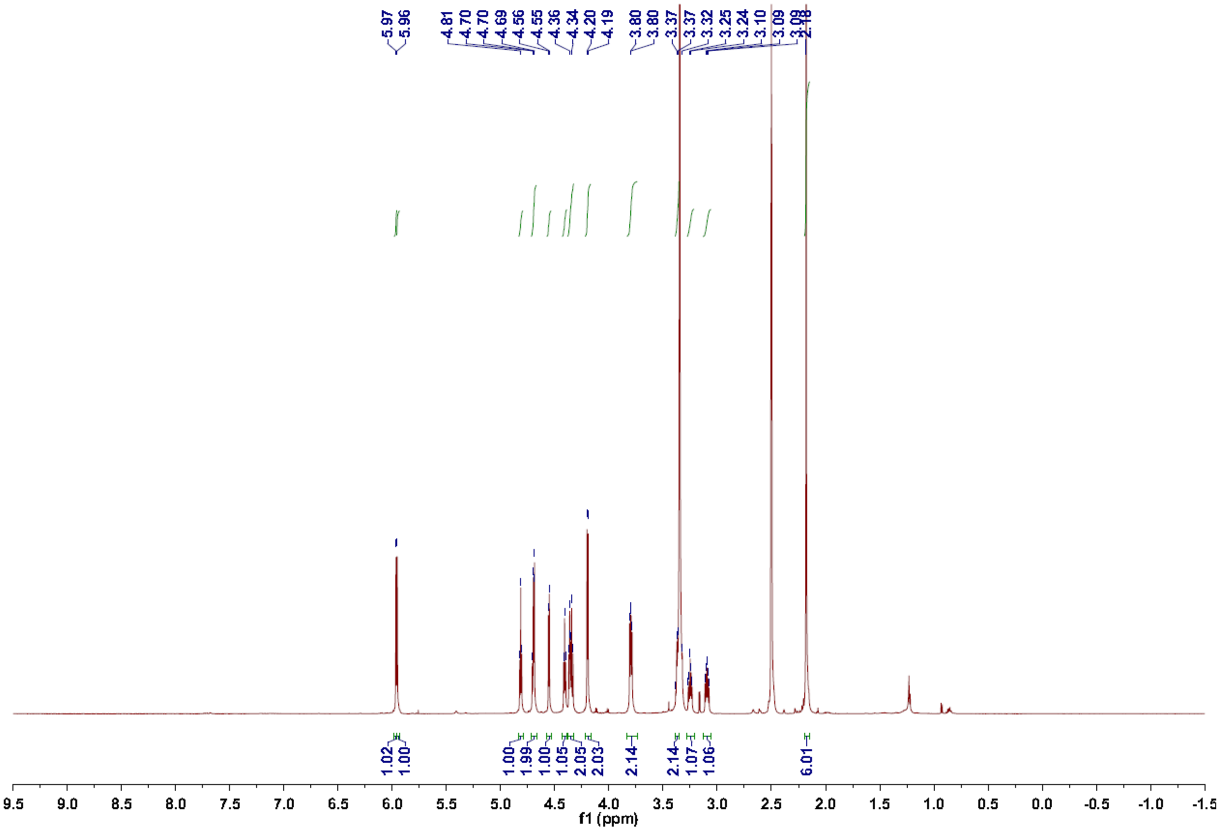

Figure S1. ^{1}H NMR spectrum of **1** in DMSO-d_6 (600 MHz)

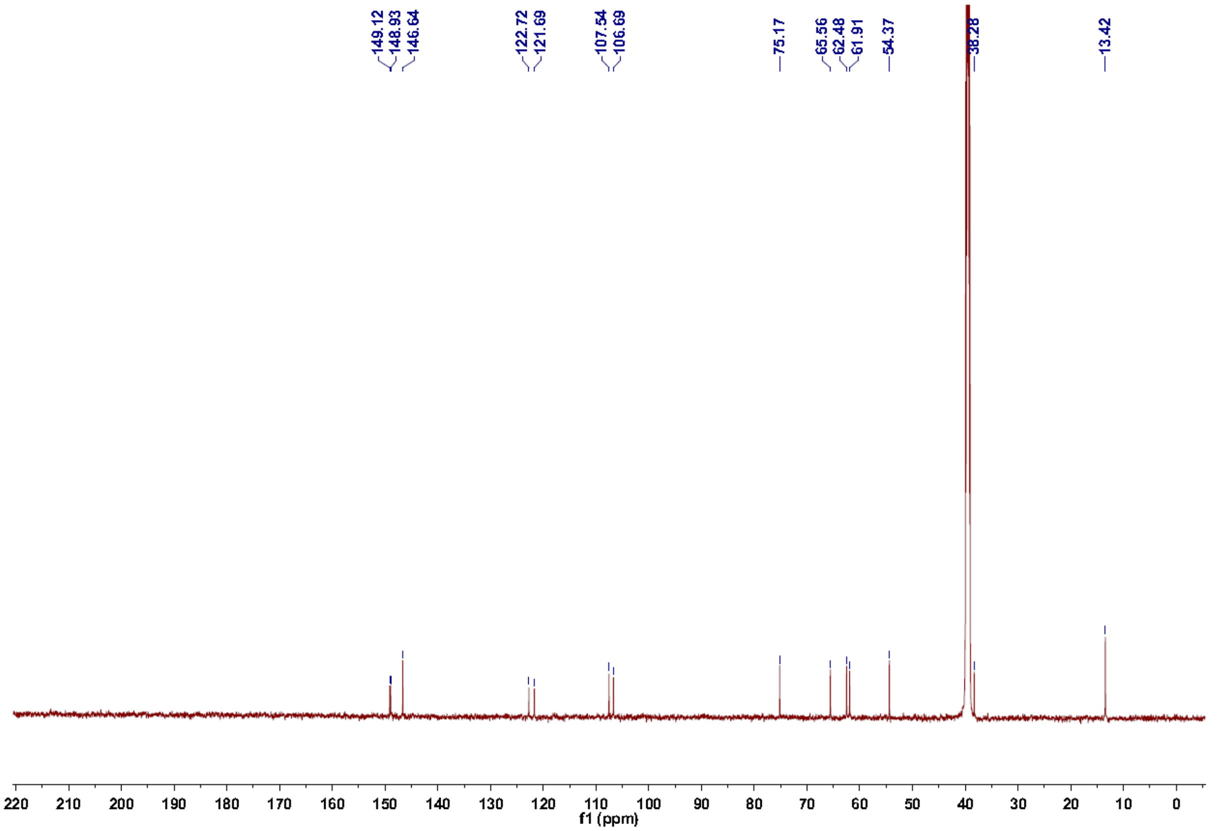

Figure S2. ^{13}C NMR spectrum of **1** in DMSO-d_6 (125 MHz)

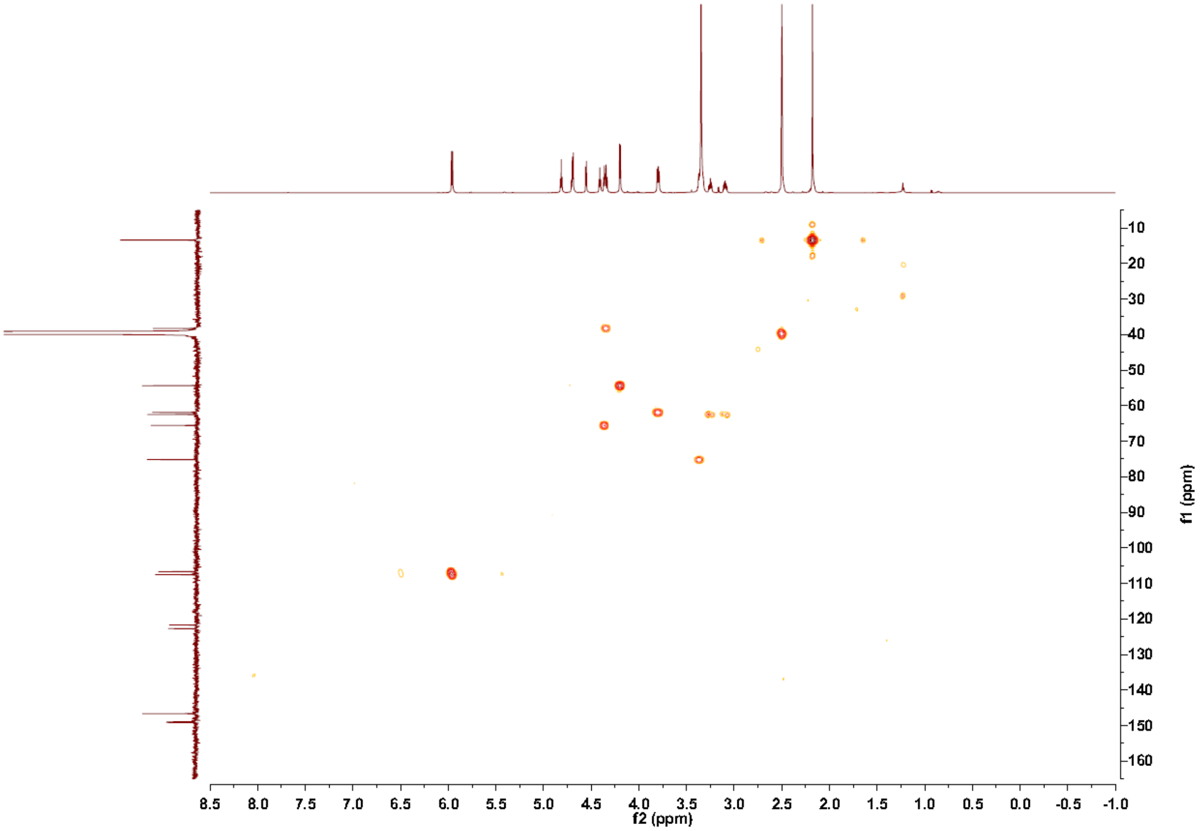

Figure S3. HSQC spectrum of **1** recorded in DMSO-d_6

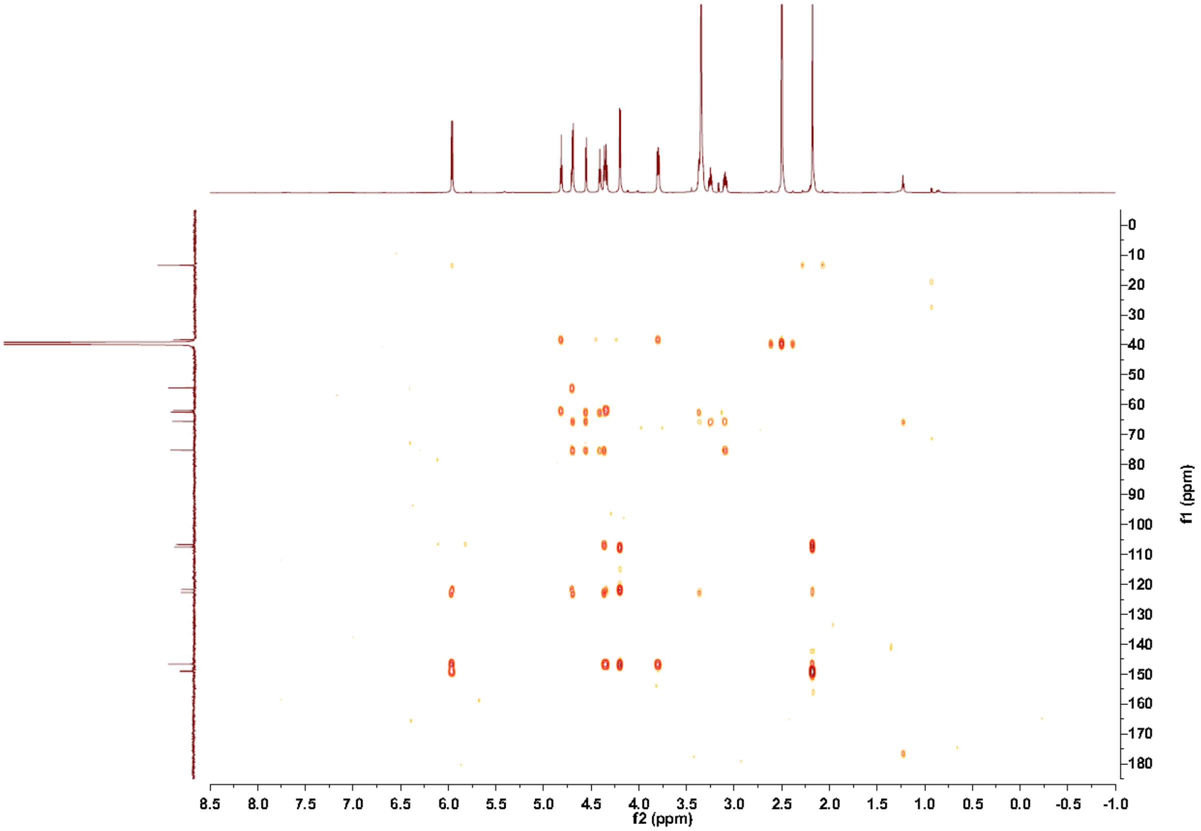

Figure S4. HMBC spectrum of **1** recorded in DMSO-d_6

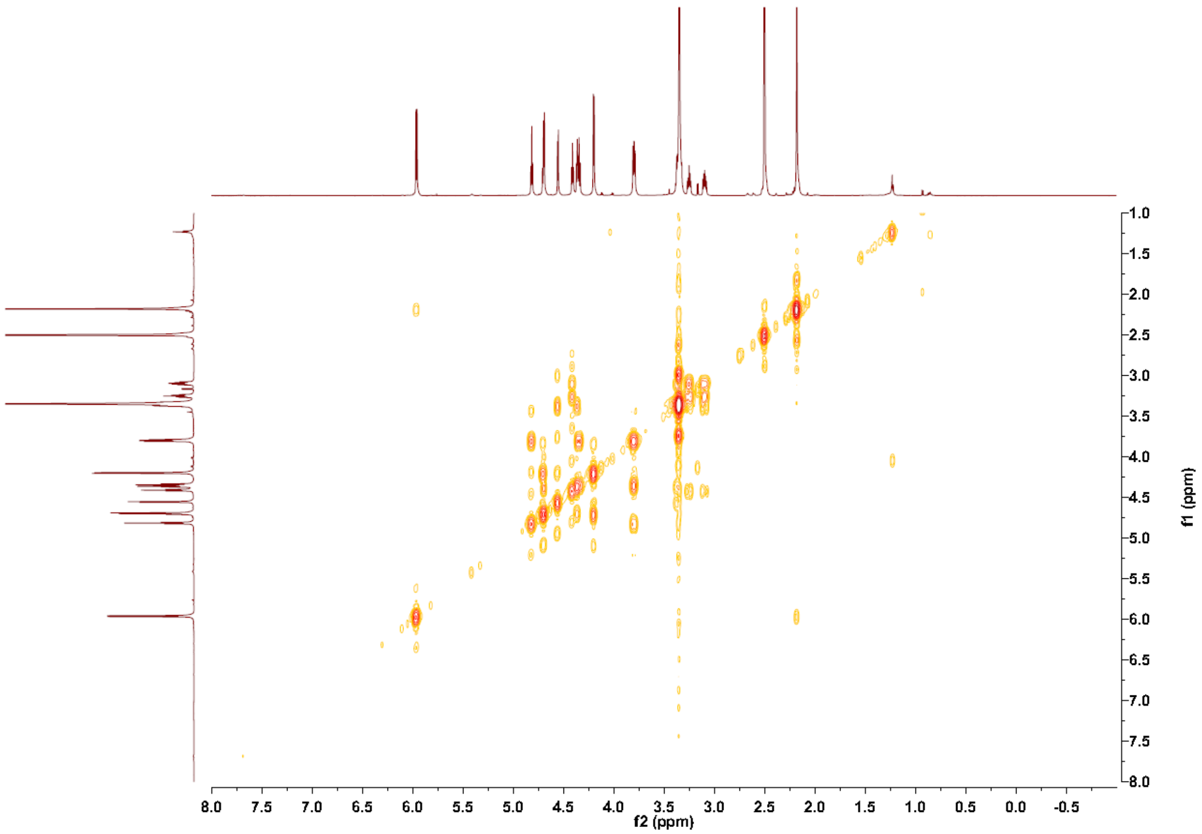

Figure S5. ^1H-^1H COSY spectrum of **1** recorded in DMSO-d_6

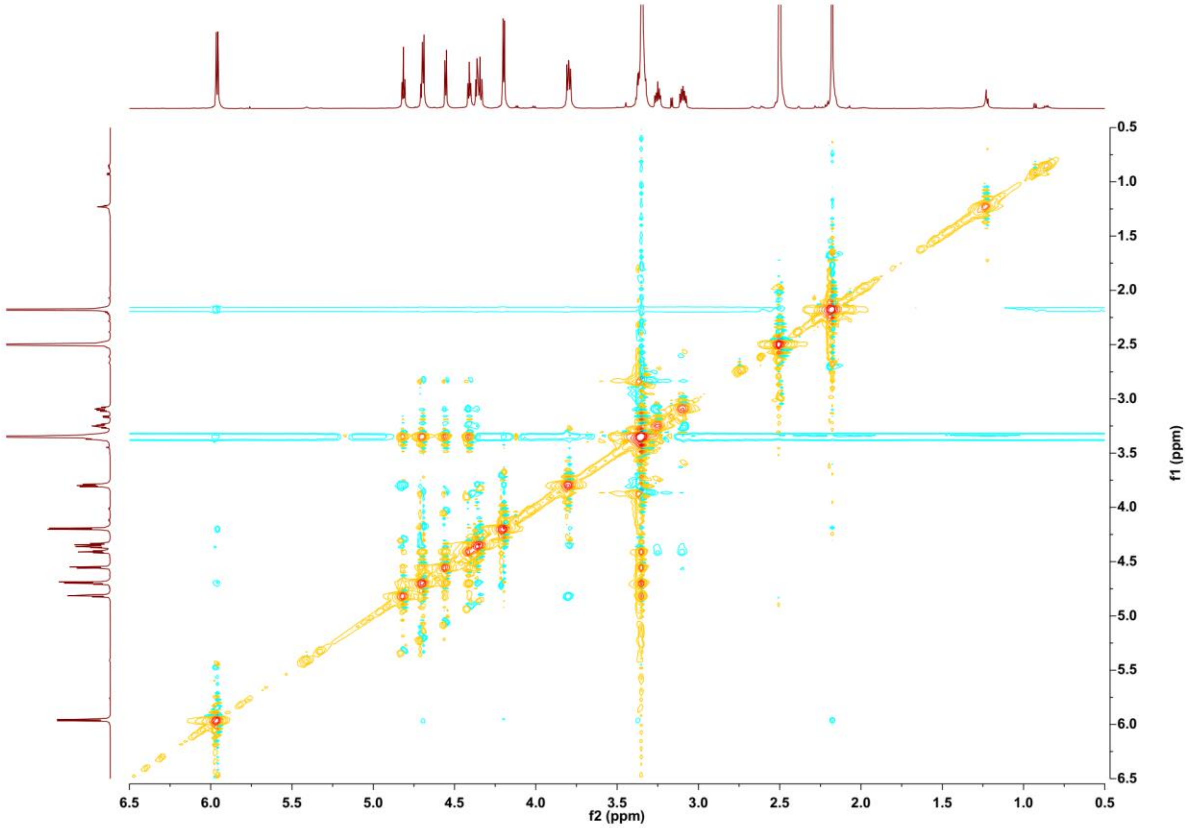

Figure S6. NOESY spectrum of **1** recorded in DMSO-d_6

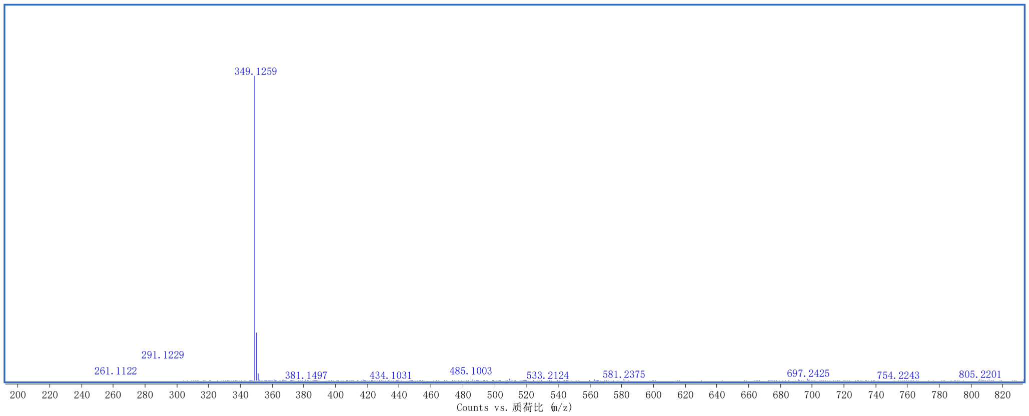

Figure S7. HRESIMS spectrum of **1**

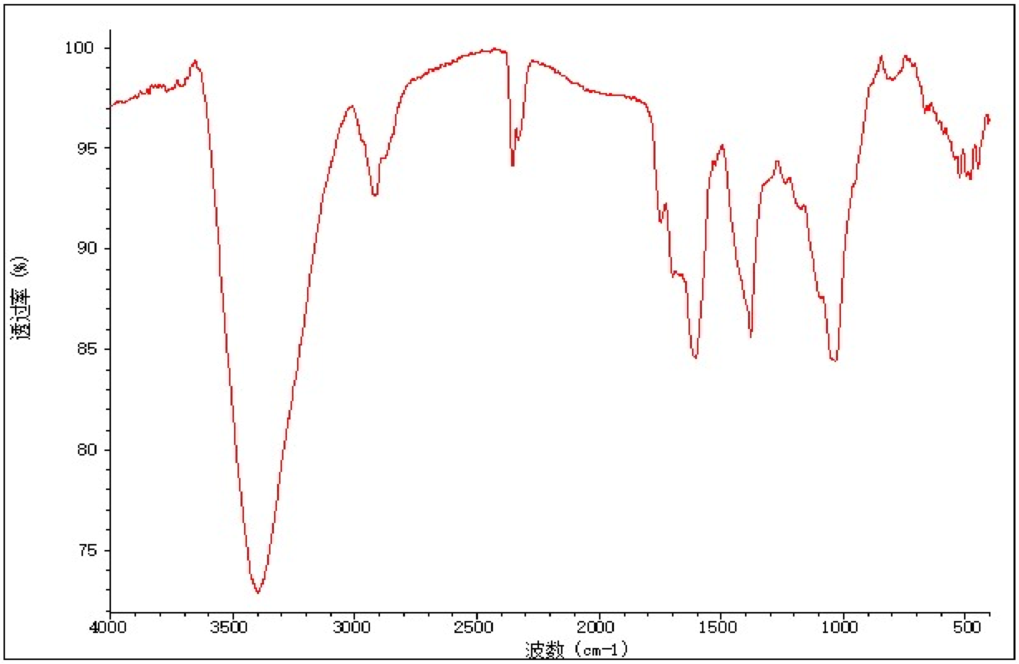

Figure S8. IR spectrum of **1**

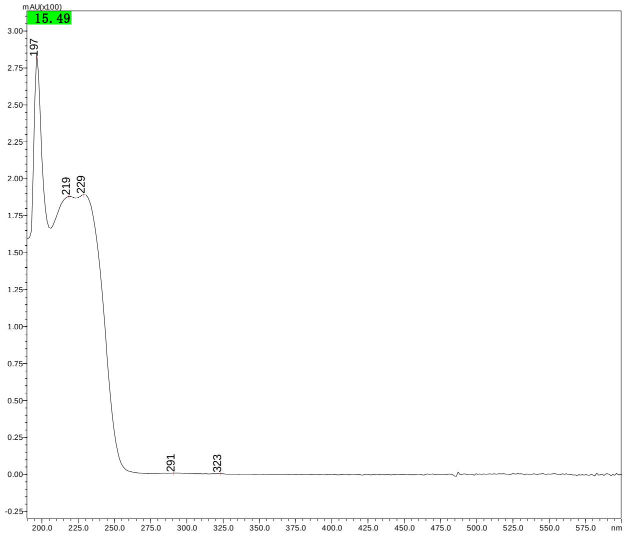

Figure S9. UV spectrum of **1**

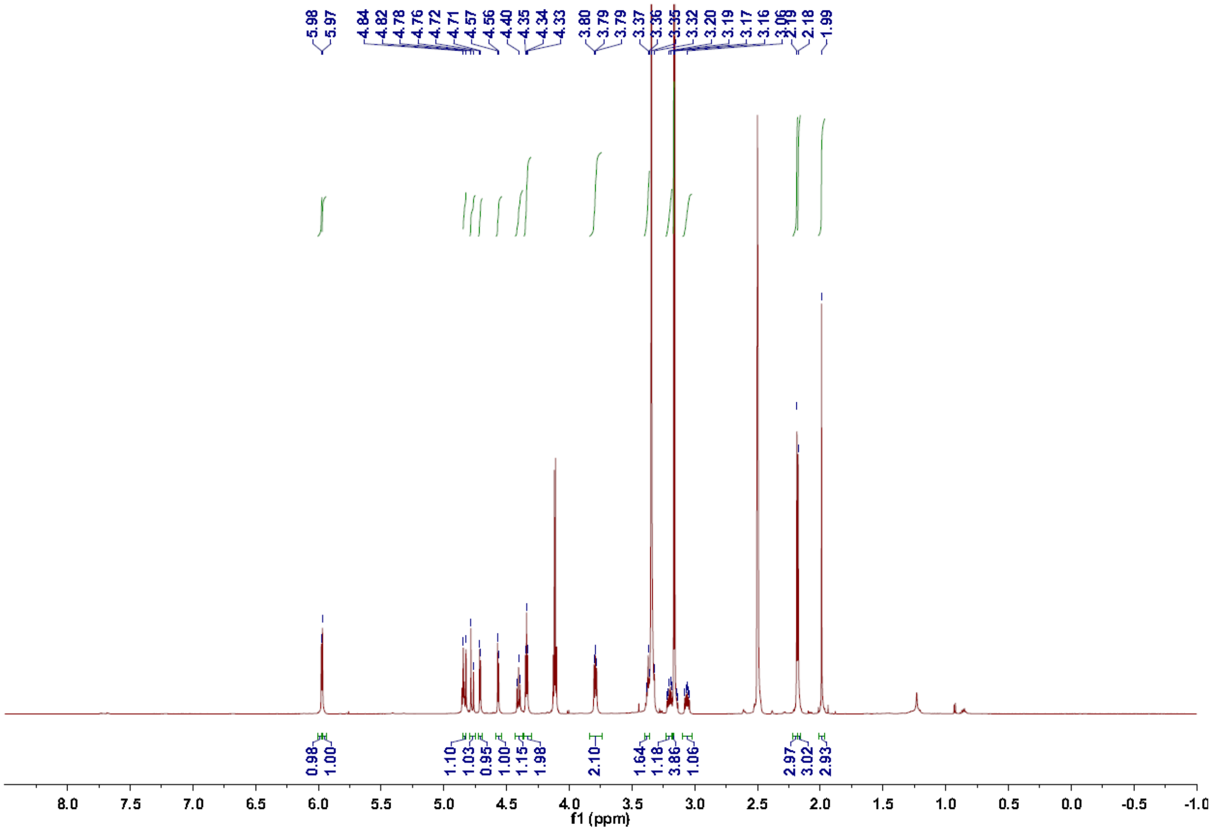

Figure S10. ^{1}H NMR spectrum of **2** in DMSO-d_6 (600 MHz)

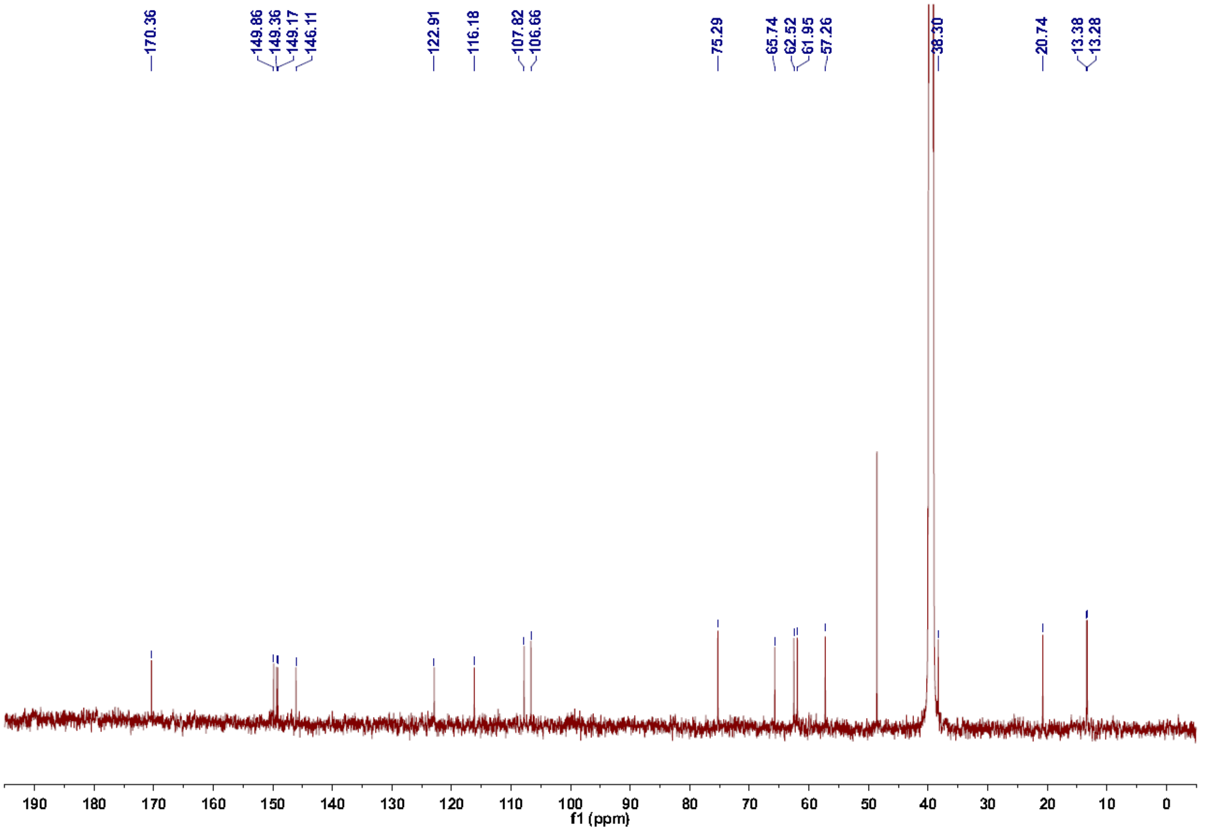

Figure S11. ^{13}C NMR spectrum of **2** in DMSO-d_6 (150 MHz)

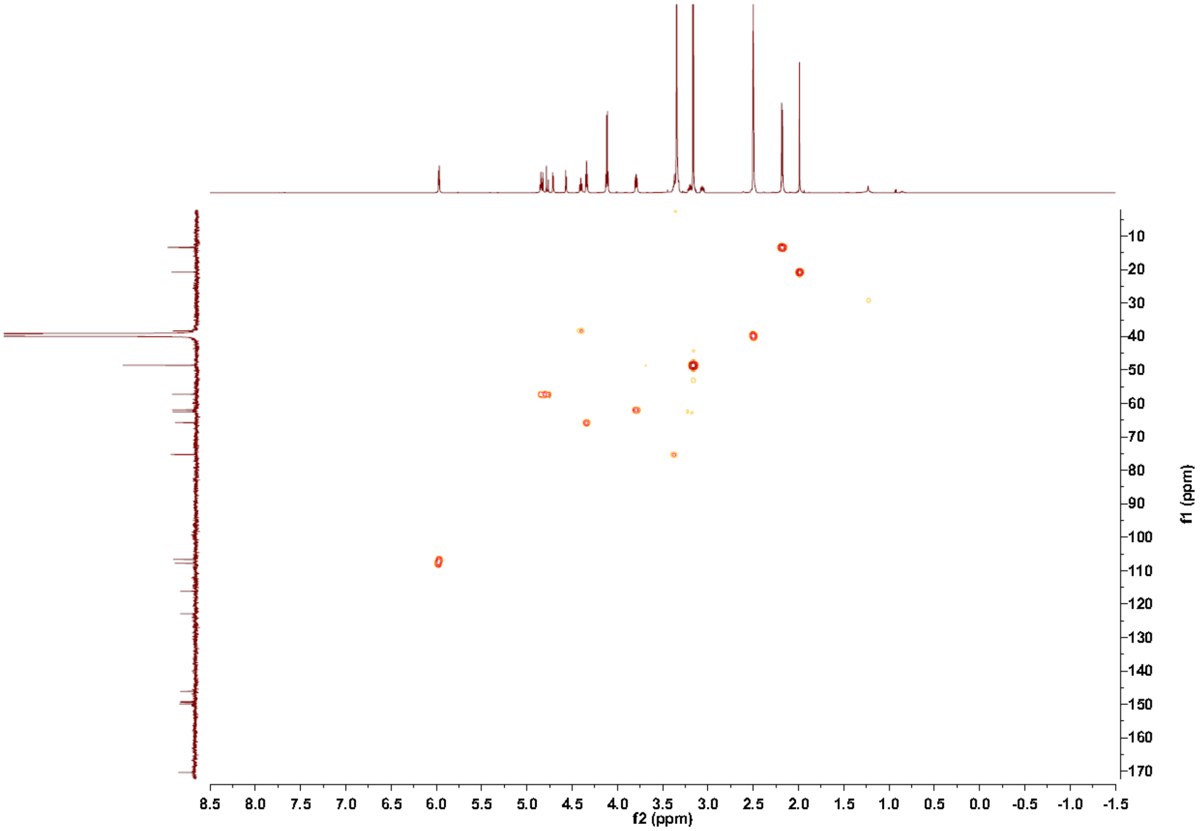

Figure S12. HSQC spectrum of **2** recorded in DMSO-d_6 (600 MHz)

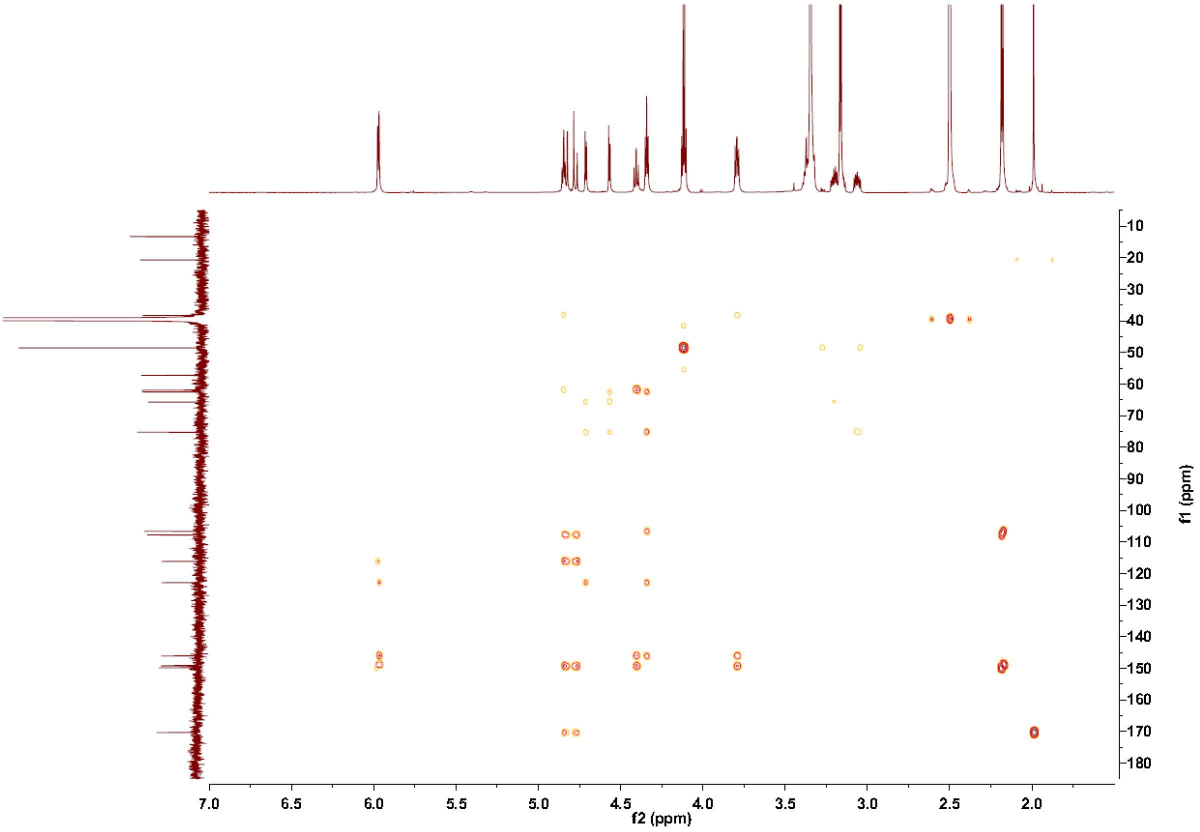

Figure S13. HMBC spectrum of **2** recorded in DMSO-d_6 (600 MHz)

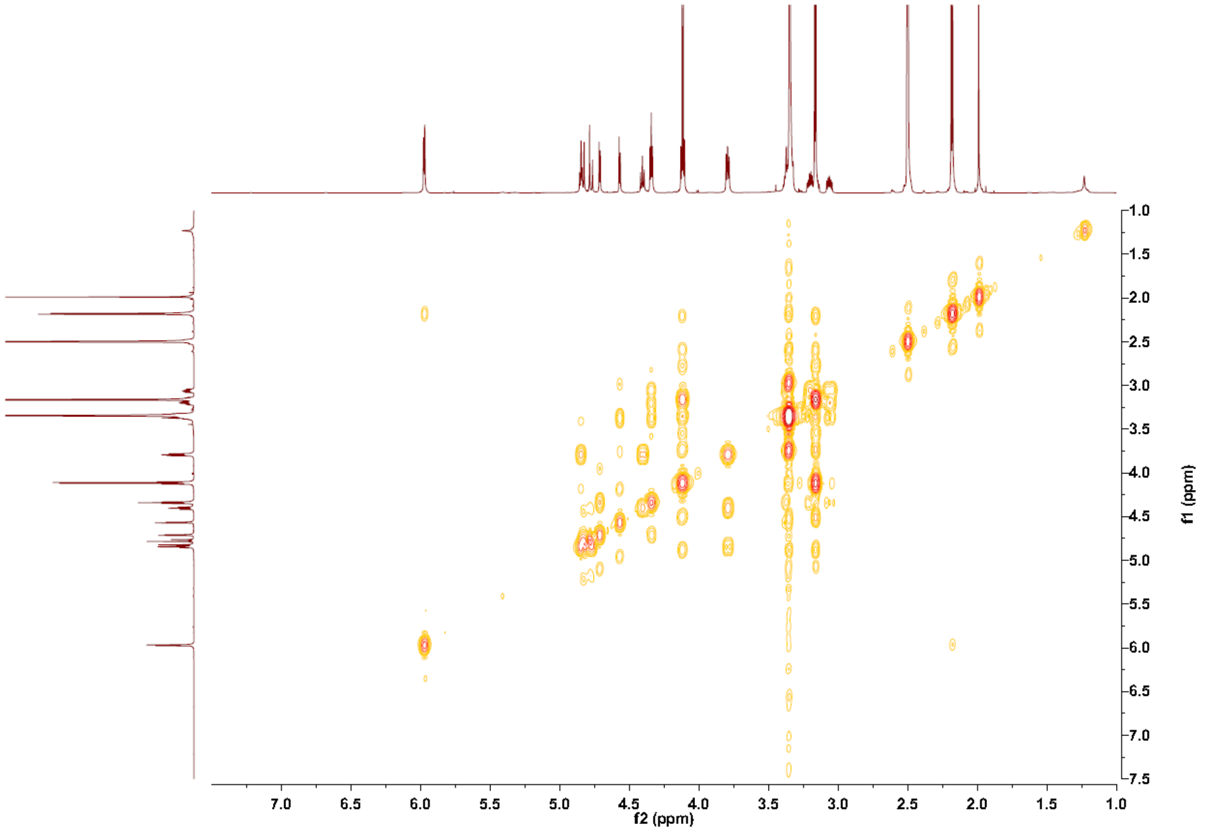

Figure S14. ^{1}H-^{1}H COSY spectrum of **2** recorded in DMSO-d_6 (600 MHz)

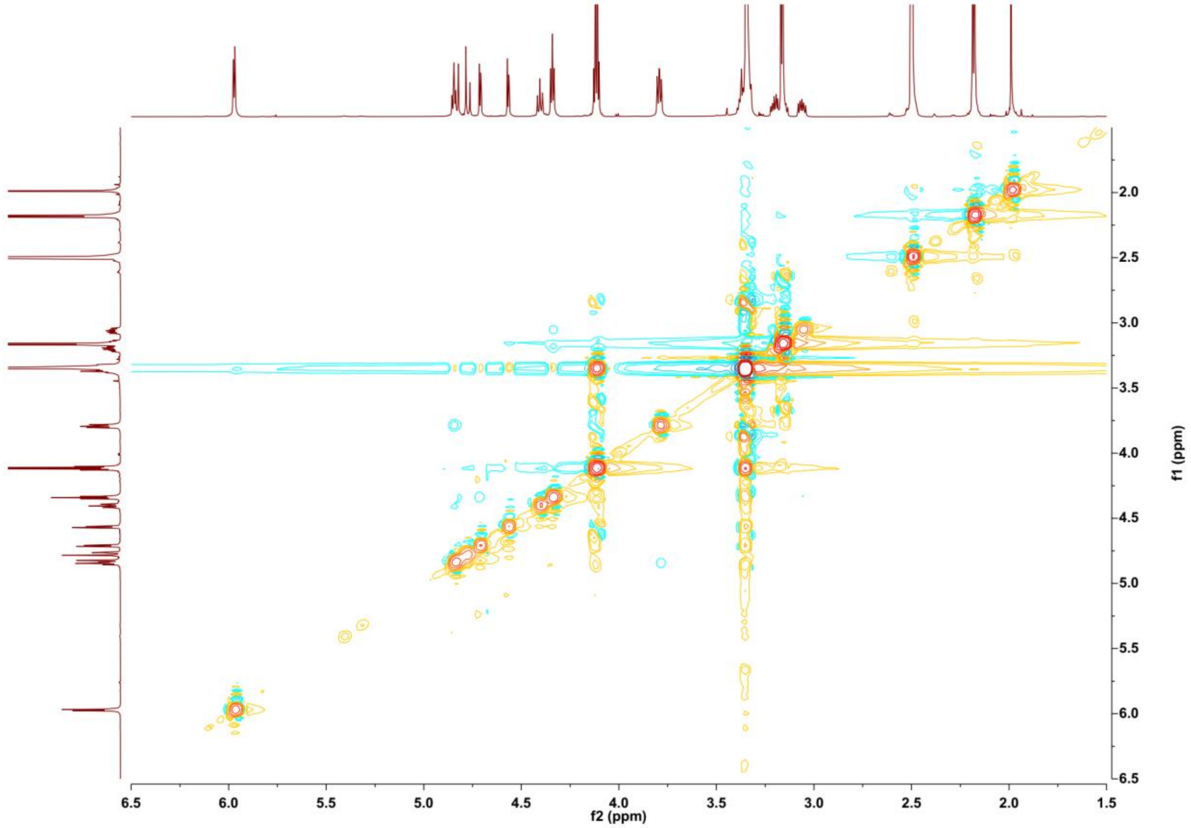

Figure S15. NOESY spectrum of **2** recorded in DMSO-d_6 (600 MHz)

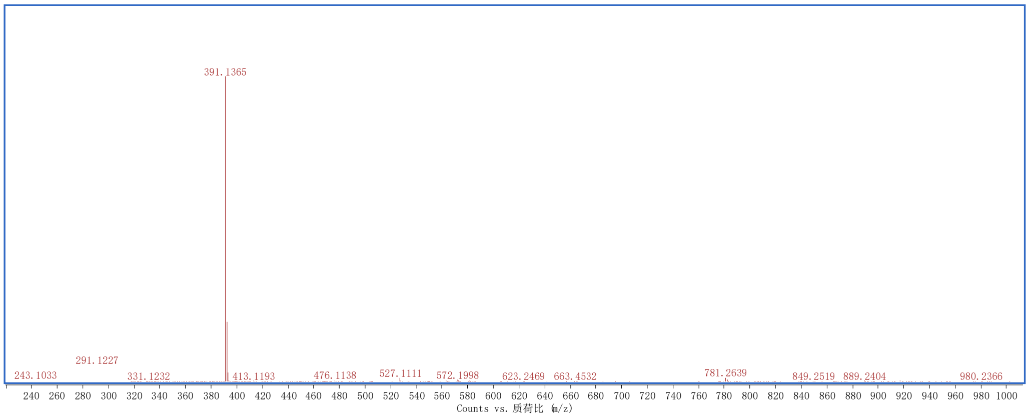

Figure S16. HRESIMS spectrum of **2**

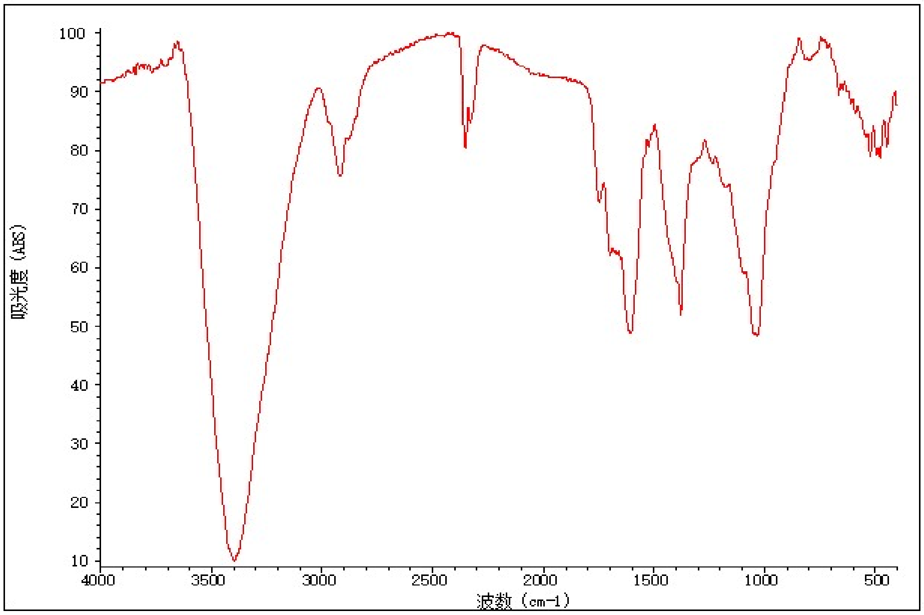

Figure S17. IR spectrum of **2**

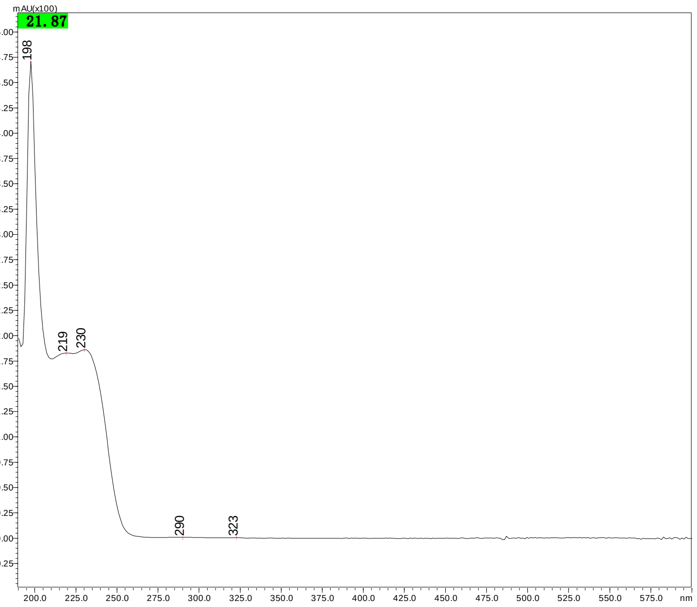

Figure S18. UV spectrum of **2**

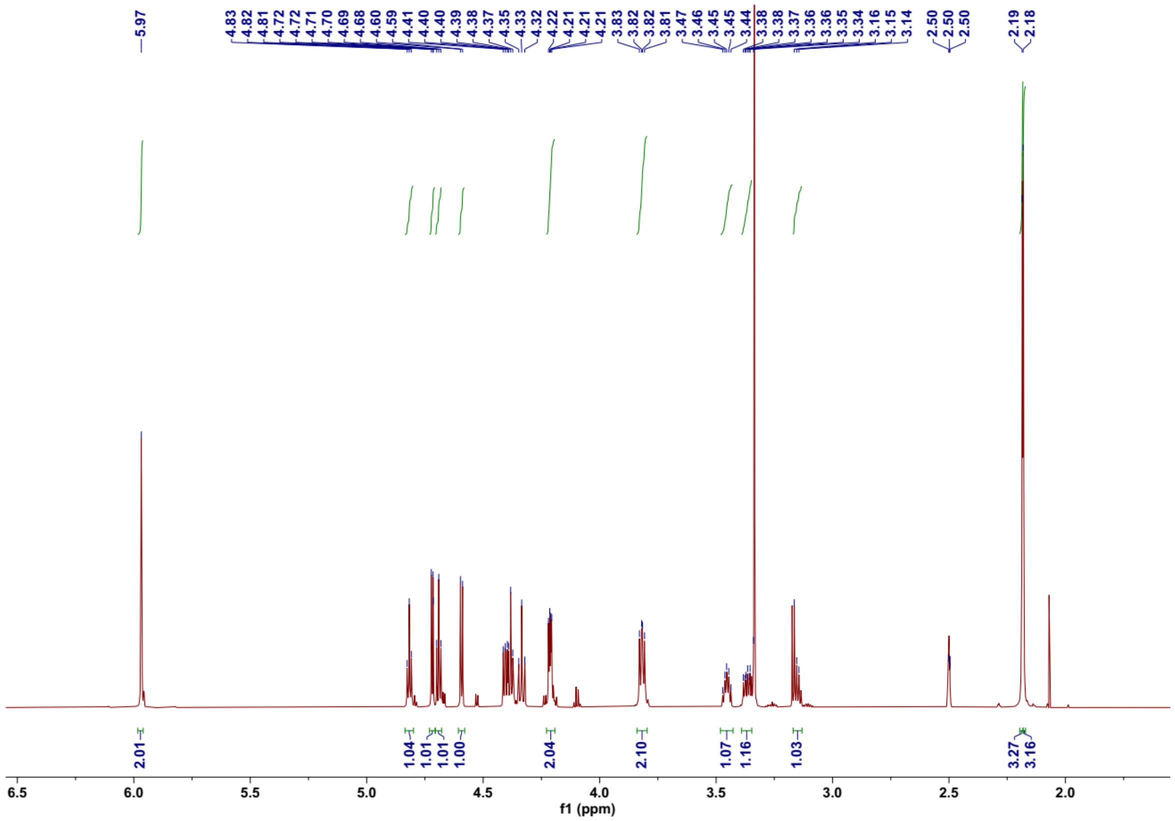

Figure S19. ^{1}H NMR spectrum of **3** in DMSO-d_6 (600 MHz)

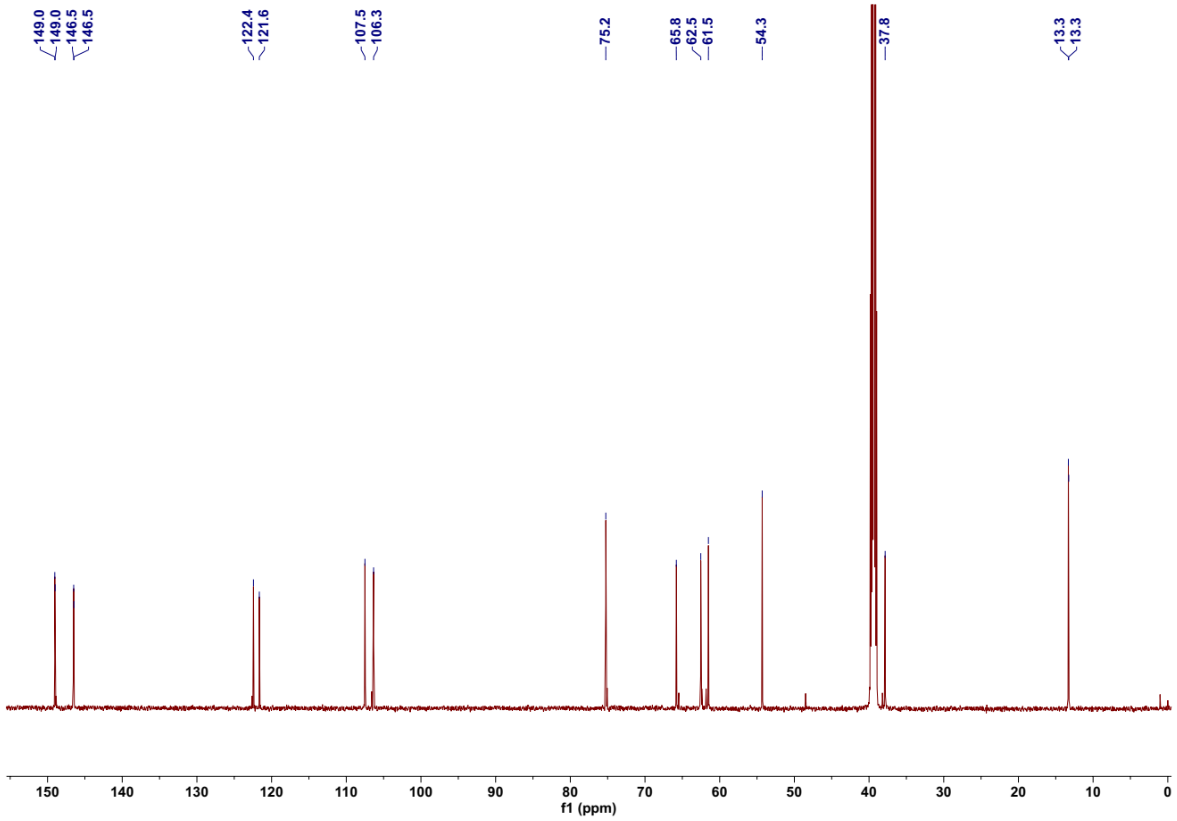

Figure S20. ^{13}C NMR spectrum of **3** in DMSO-d_6 (125 MHz)

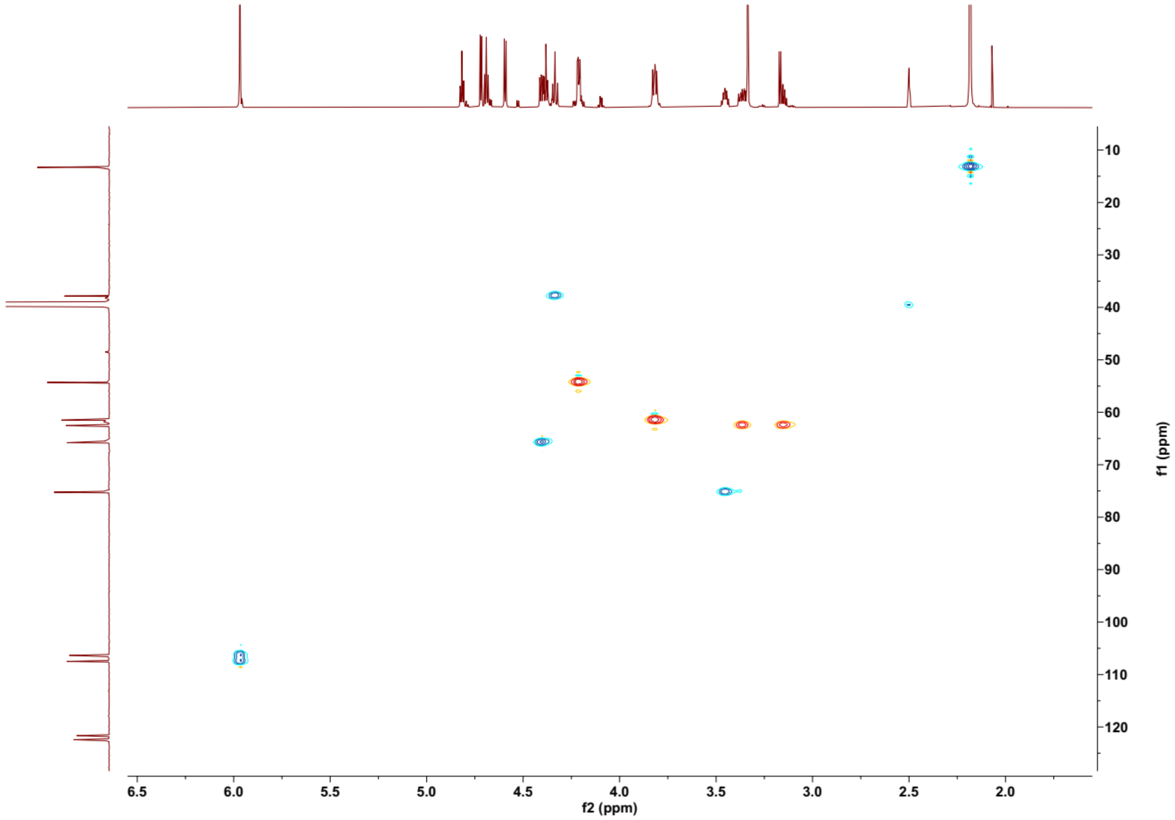

Figure S21. HSQC spectrum of **3** in DMSO-*d*₆

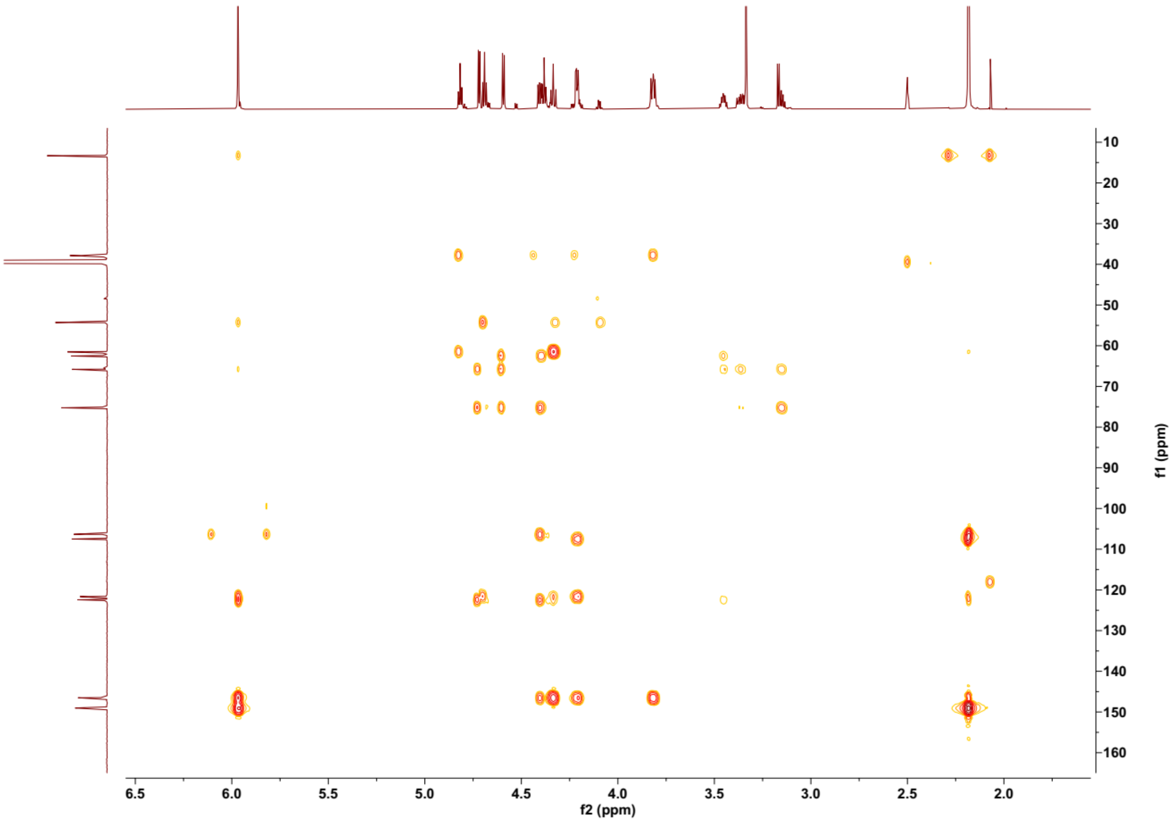

Figure S22. HMBC spectrum of **3** in DMSO-*d*₆ (600 MHz)

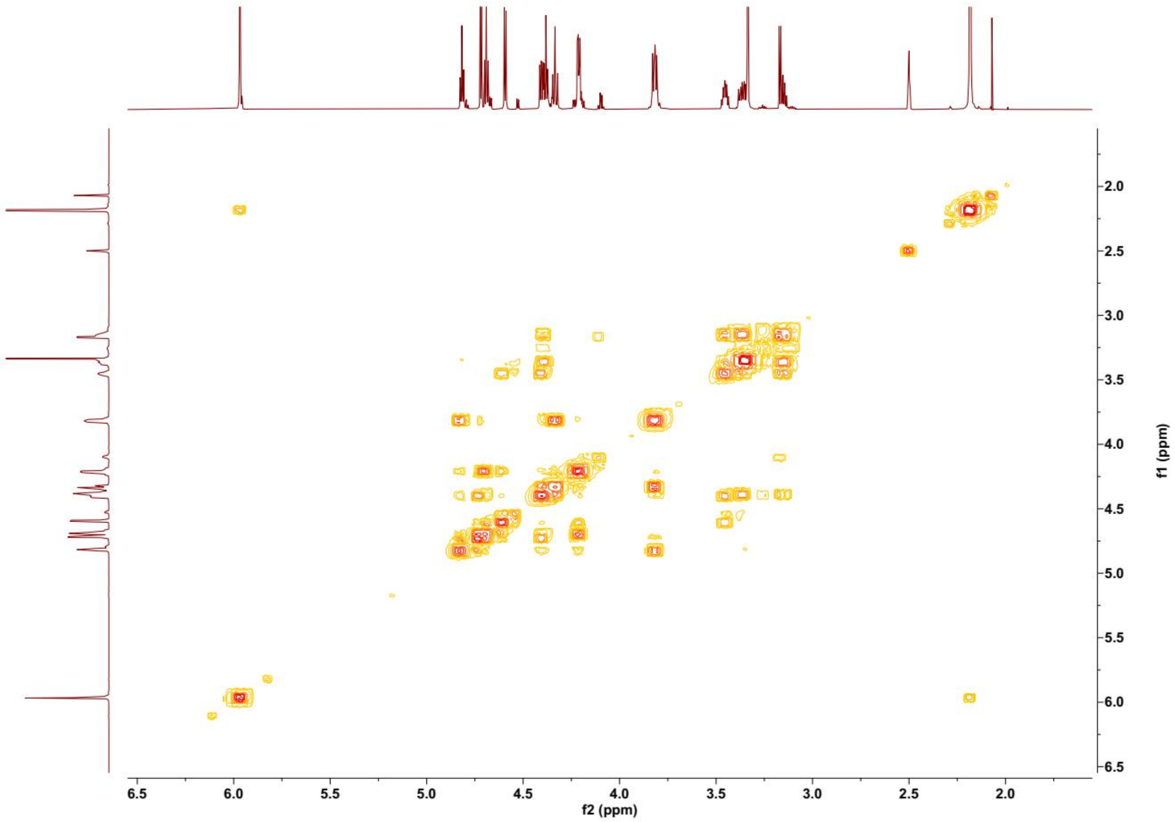

Figure S23. ^{1}H-^{1}H COSY spectrum of **3** in DMSO-d_6 (600 MHz)

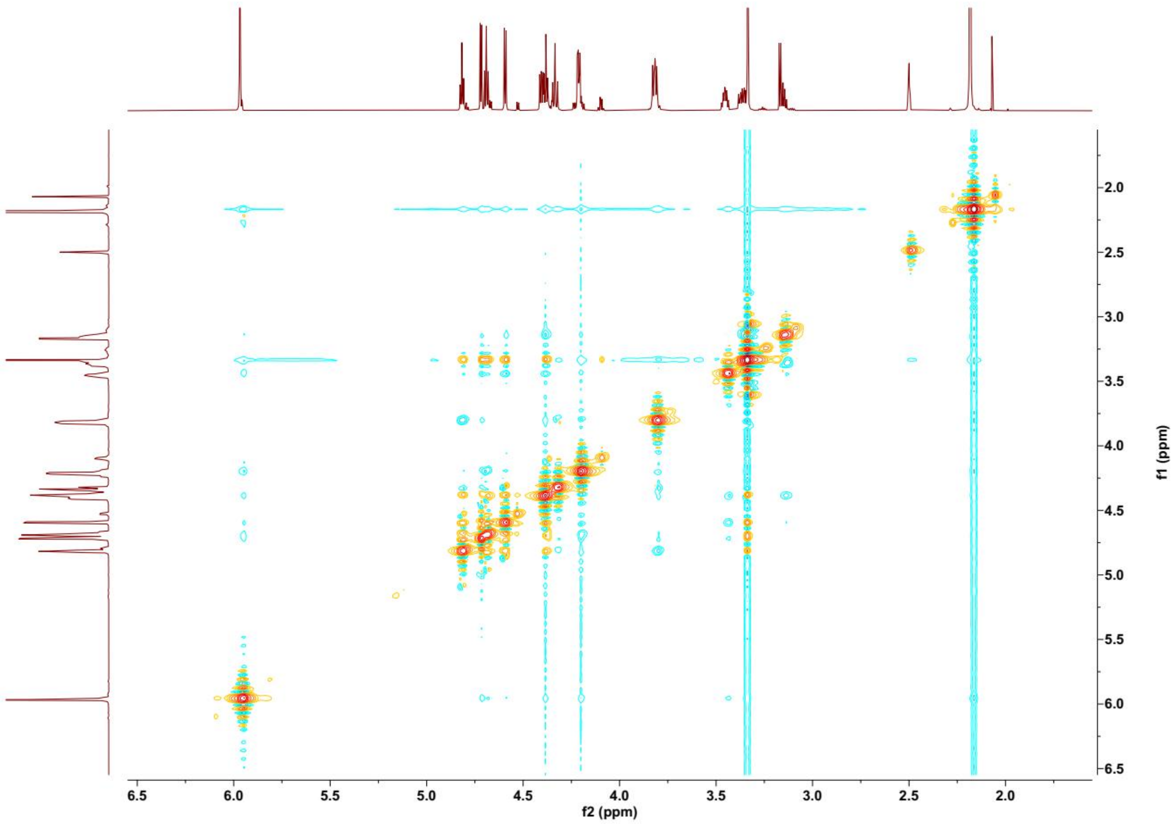

Figure S24. NOESY spectrum of **3** in DMSO-d_6 (600 MHz)

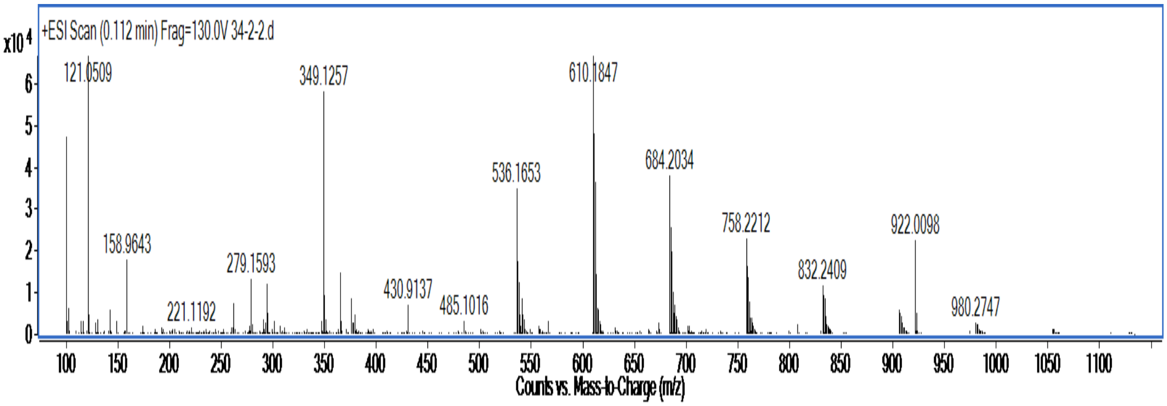

Figure S25. HRESIMS spectrum of **3**

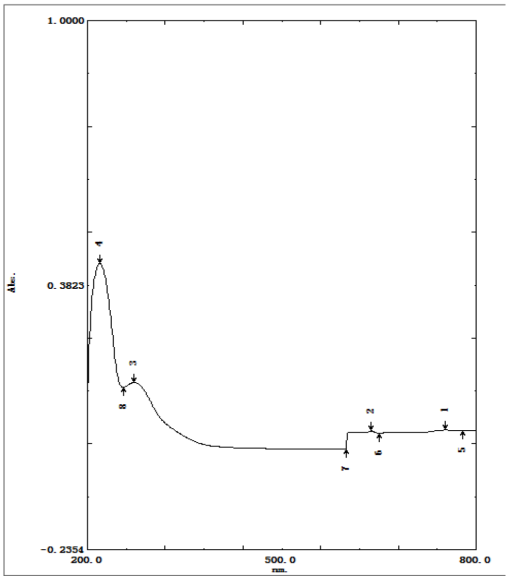

Figure S26. UV spectrum of **3**

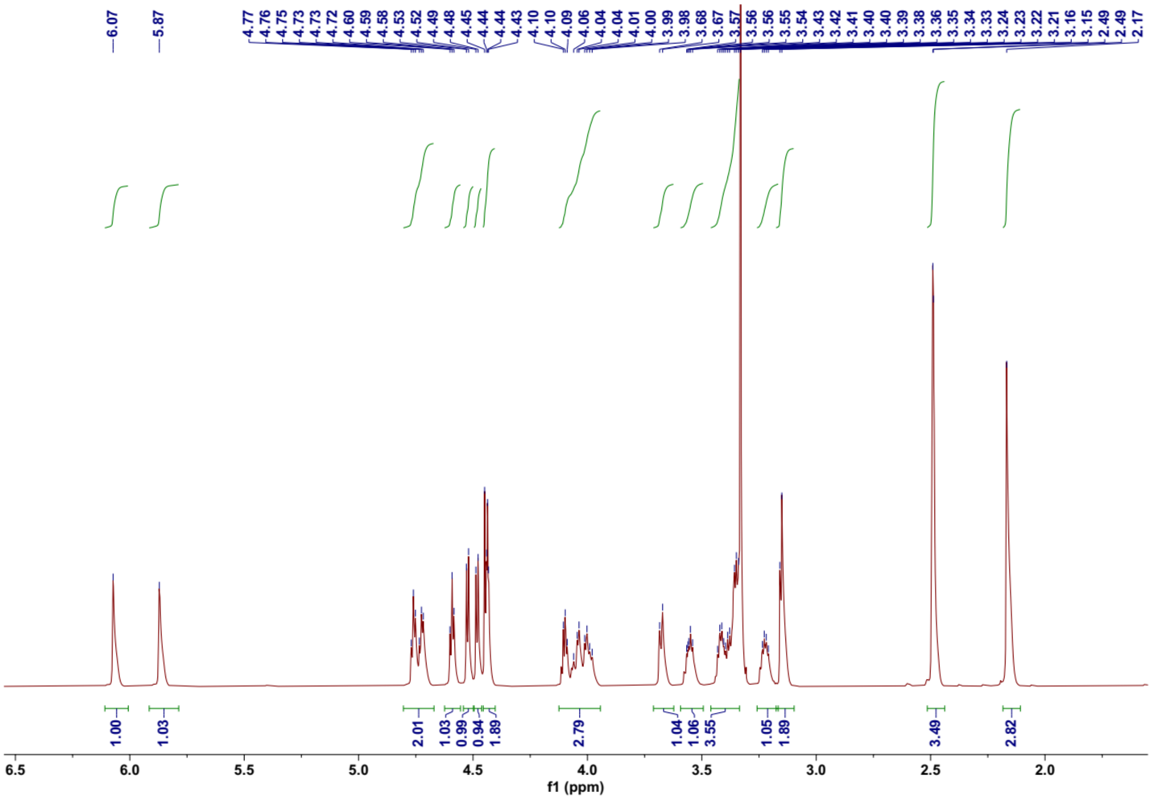

Figure S27. ^{1}H NMR spectrum of **4** in DMSO-d_6 (600 MHz)

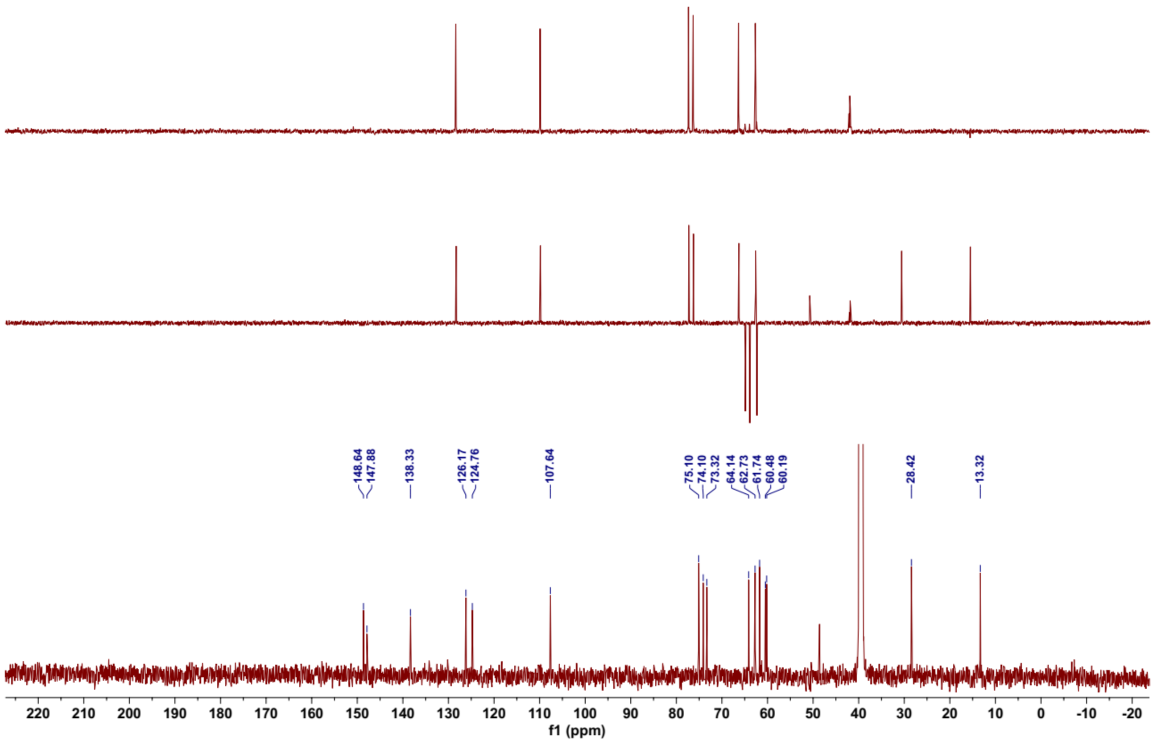

Figure S28. ^{13}C and DEPT NMR spectra of **4** in DMSO-d_6 (125 MHz)

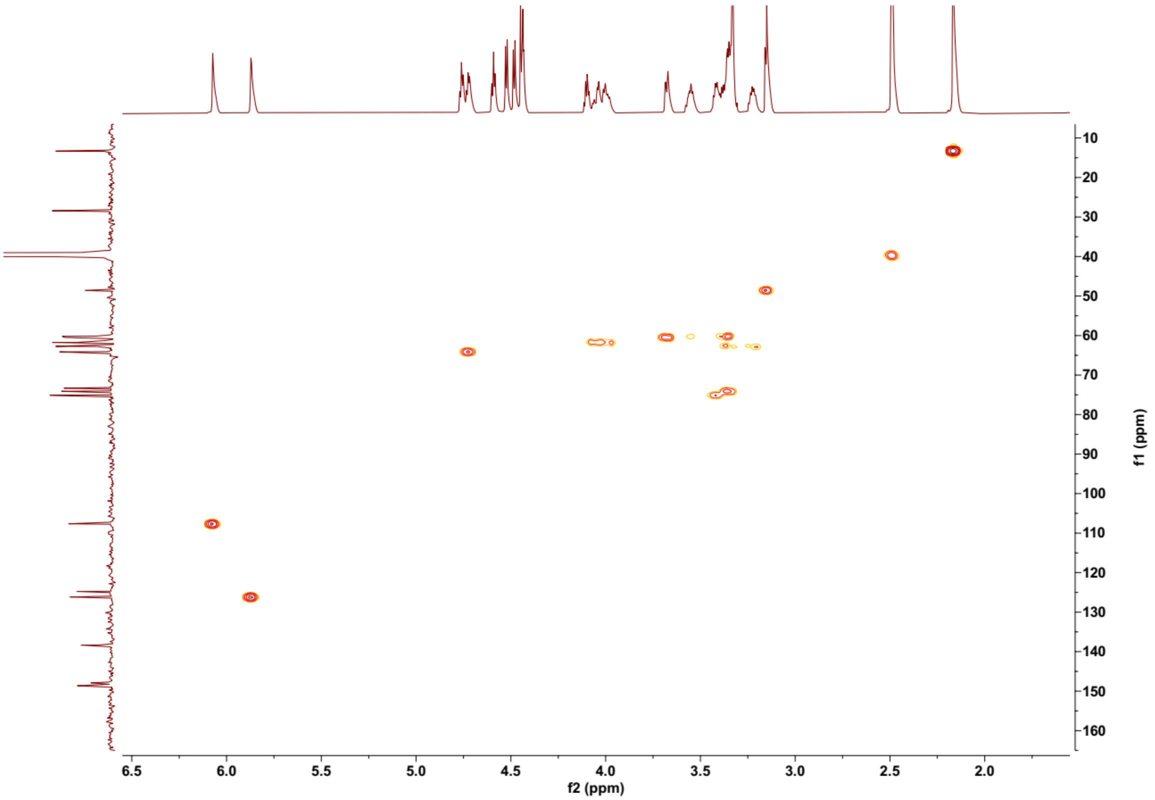

Figure S29. HSQC spectrum of **4** in DMSO-d_6

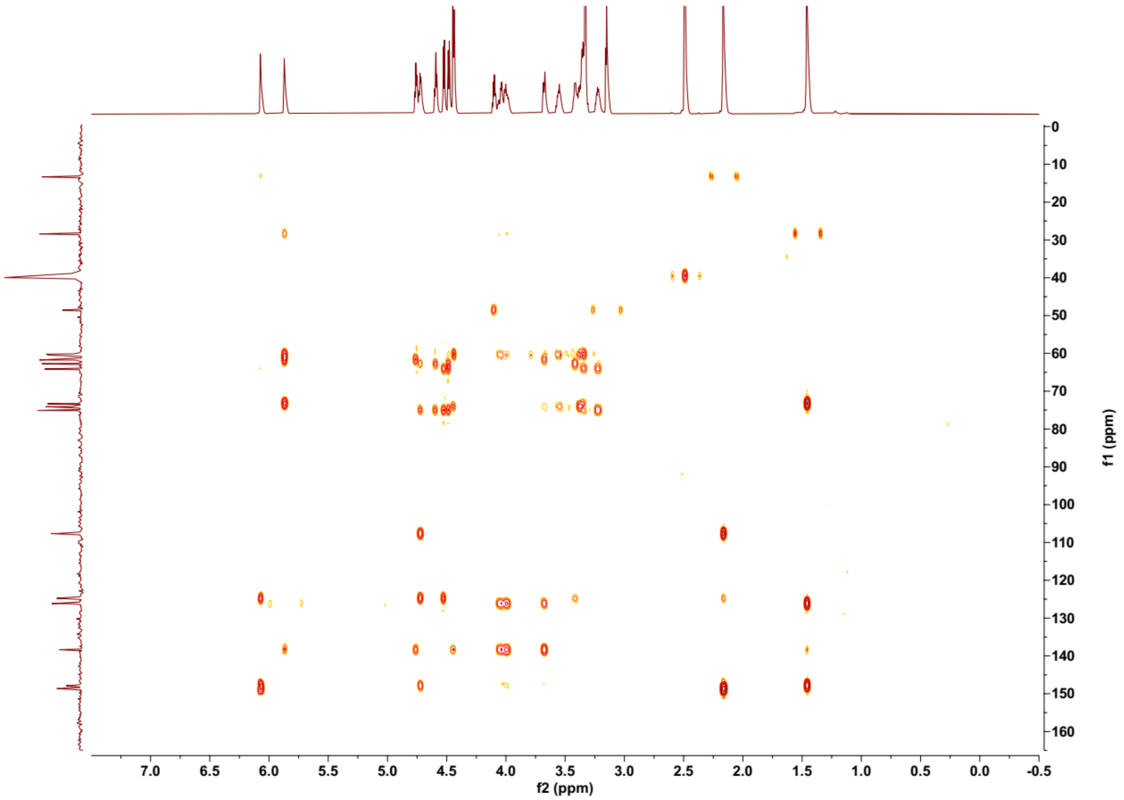

Figure S30. HMBC spectrum of **4** in DMSO-d_6

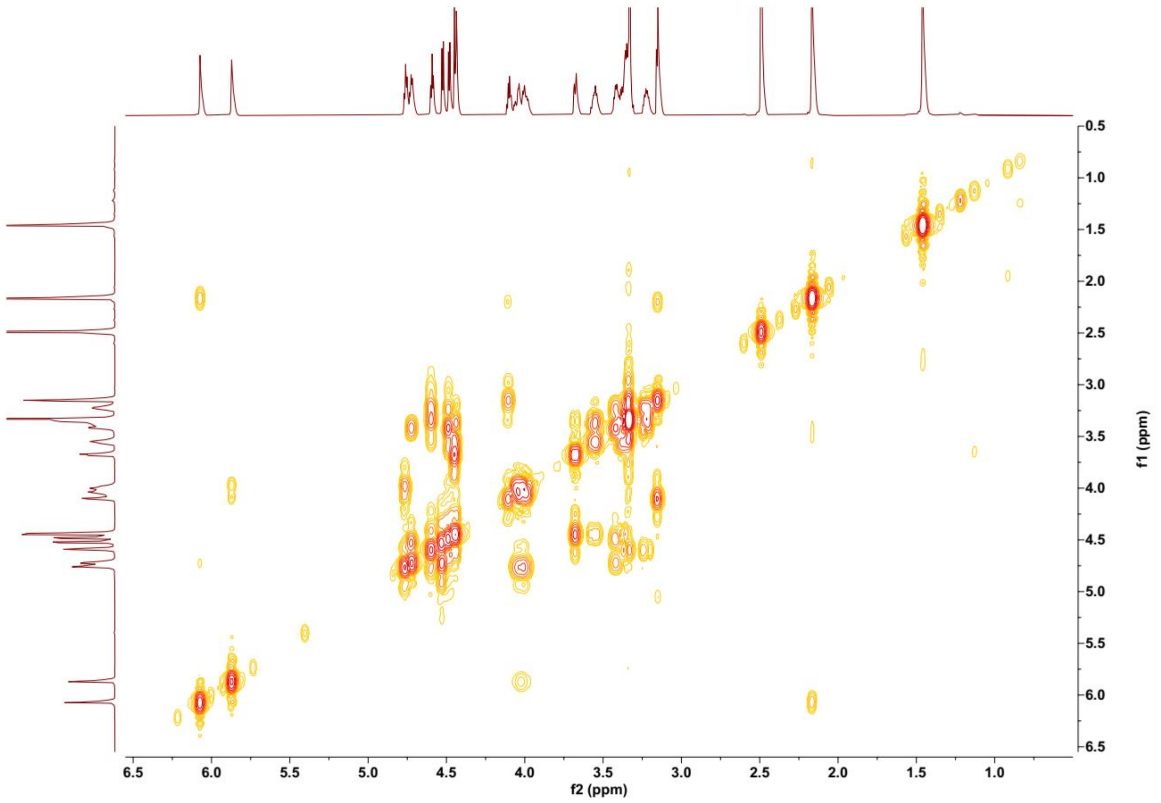

Figure S31. ^{1}H-^{1}H COSY spectrum of **4** in DMSO-d_6

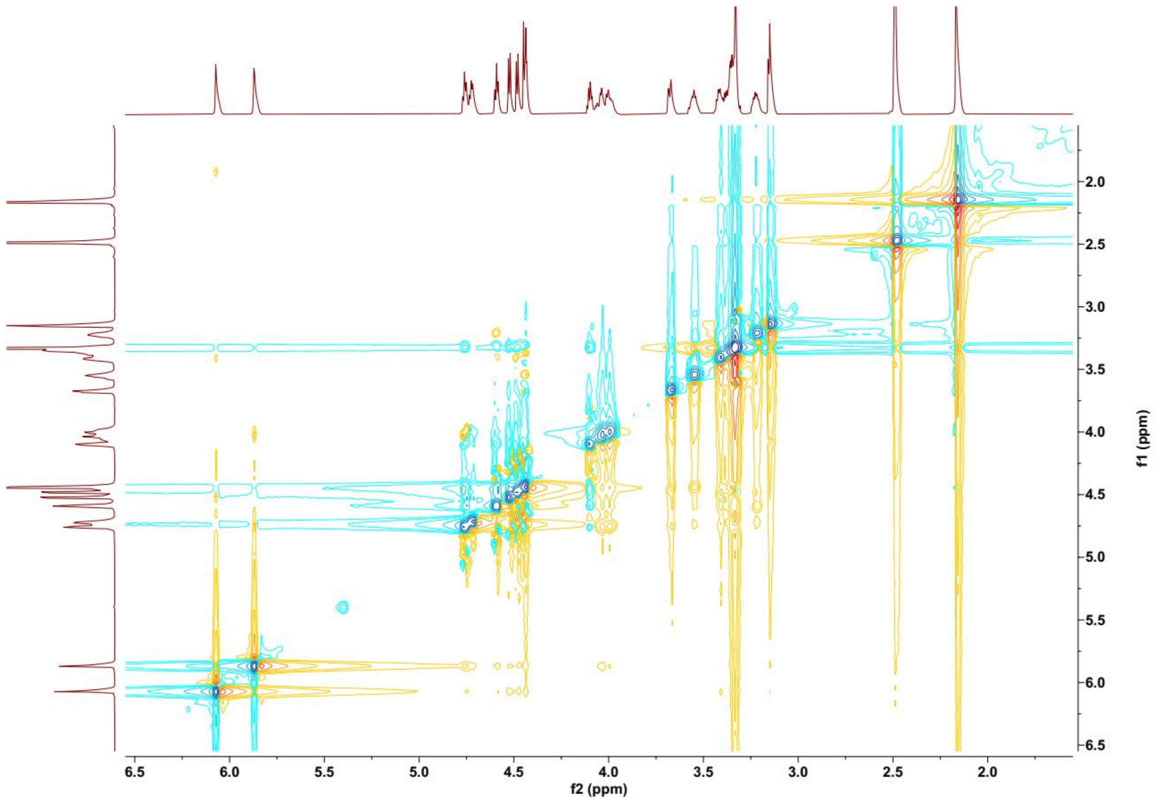

Figure S32. NOESY spectrum of **4** in DMSO-d_6

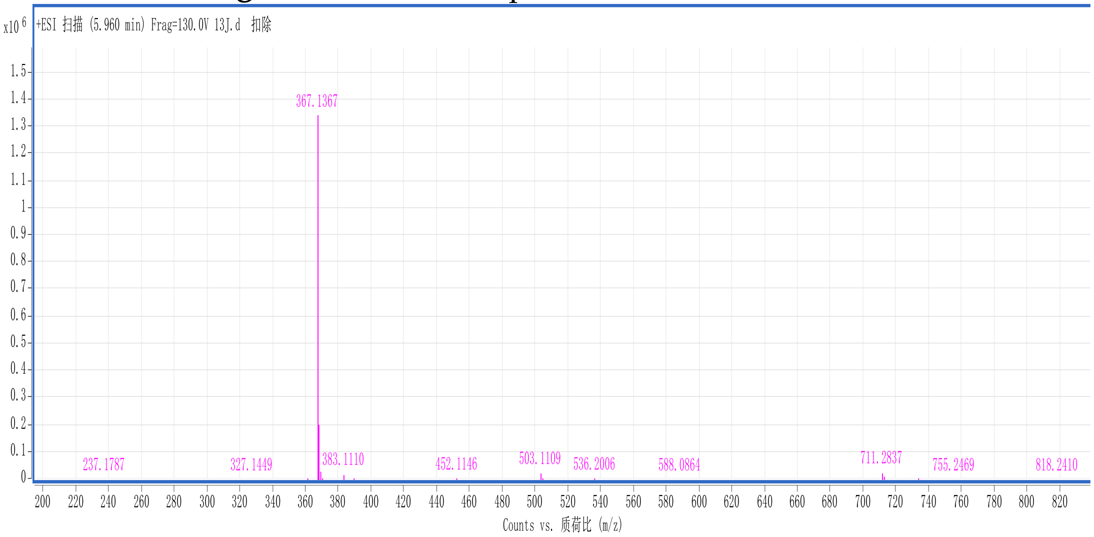

Figure S33. HRESIMS spectrum of **4**

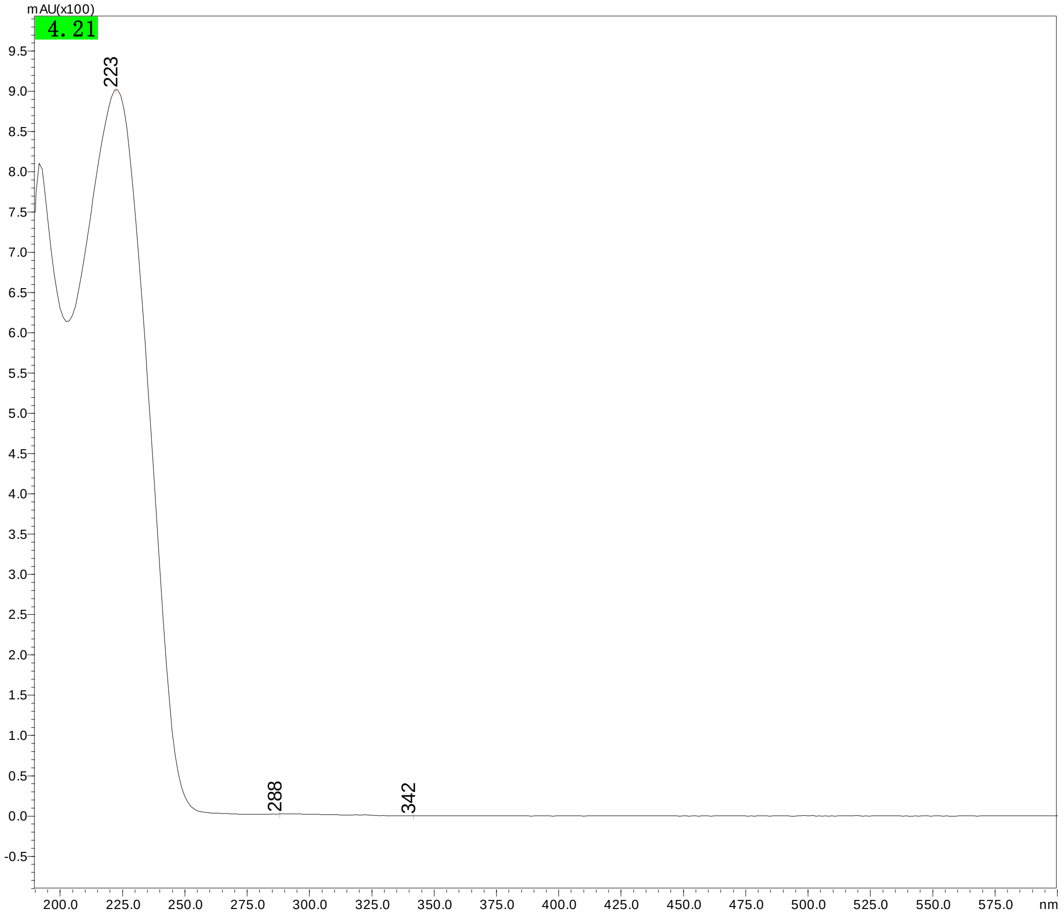

Figure S34. UV spectrum of **4**

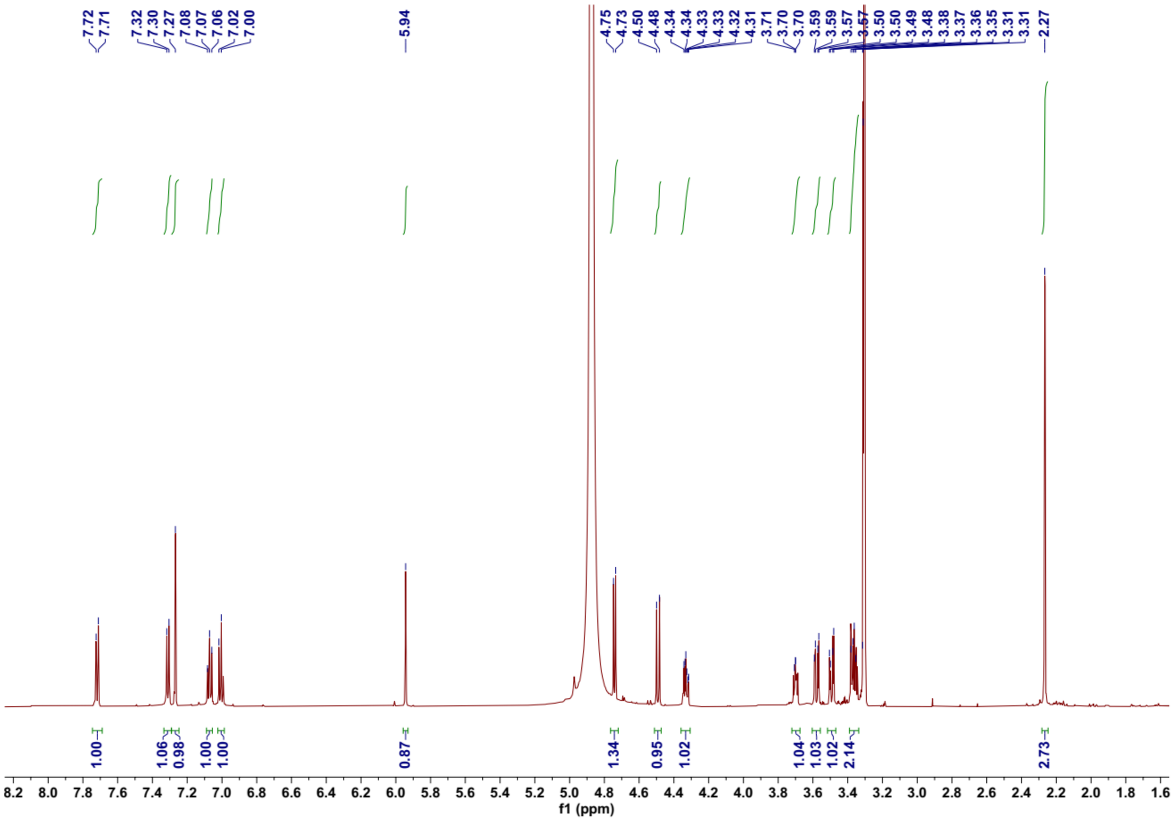

Figure S35. ^{1}H NMR spectrum of **5** in methanol-d_4 (600 MHz)

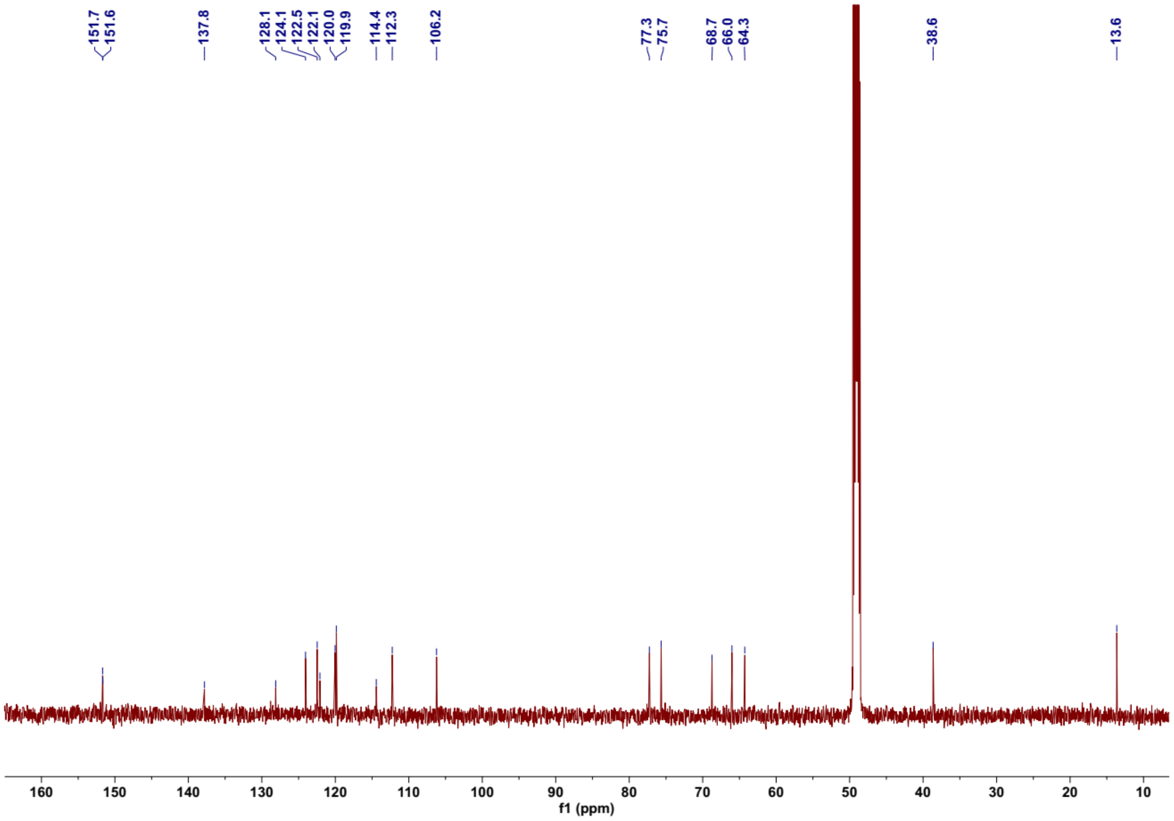

Figure S36. ^{13}C NMR spectrum of **5** in methanol-*d₄* (600 MHz)

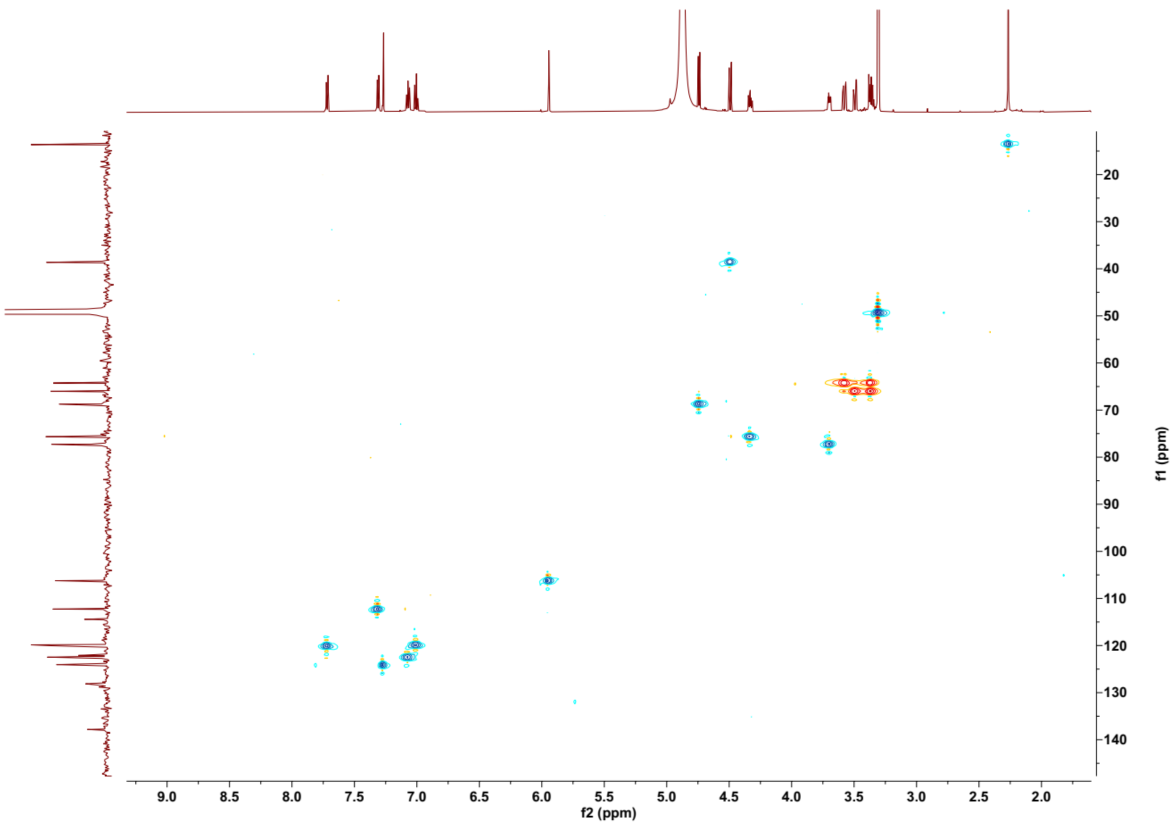

Figure S37. HSQC spectrum of **5** in methanol-d_4

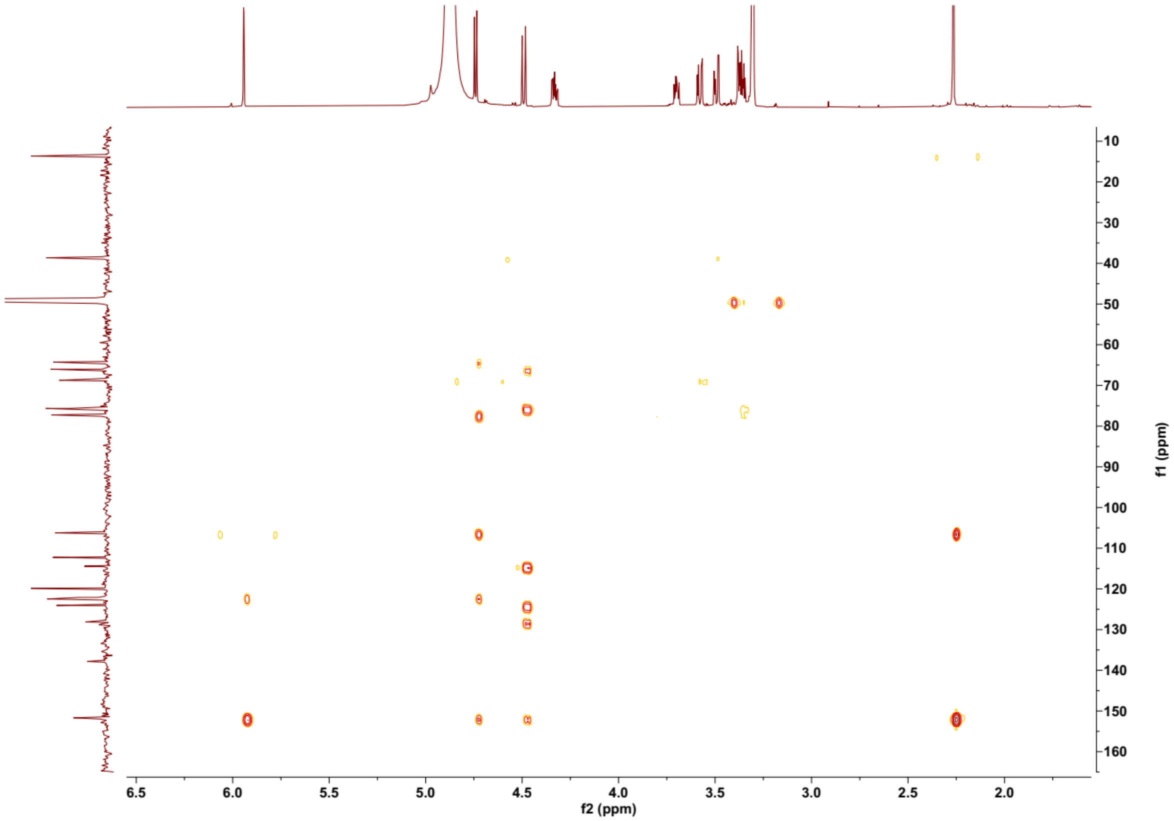

Figure S38. HMBC spectrum of **5** in methanol-d_4

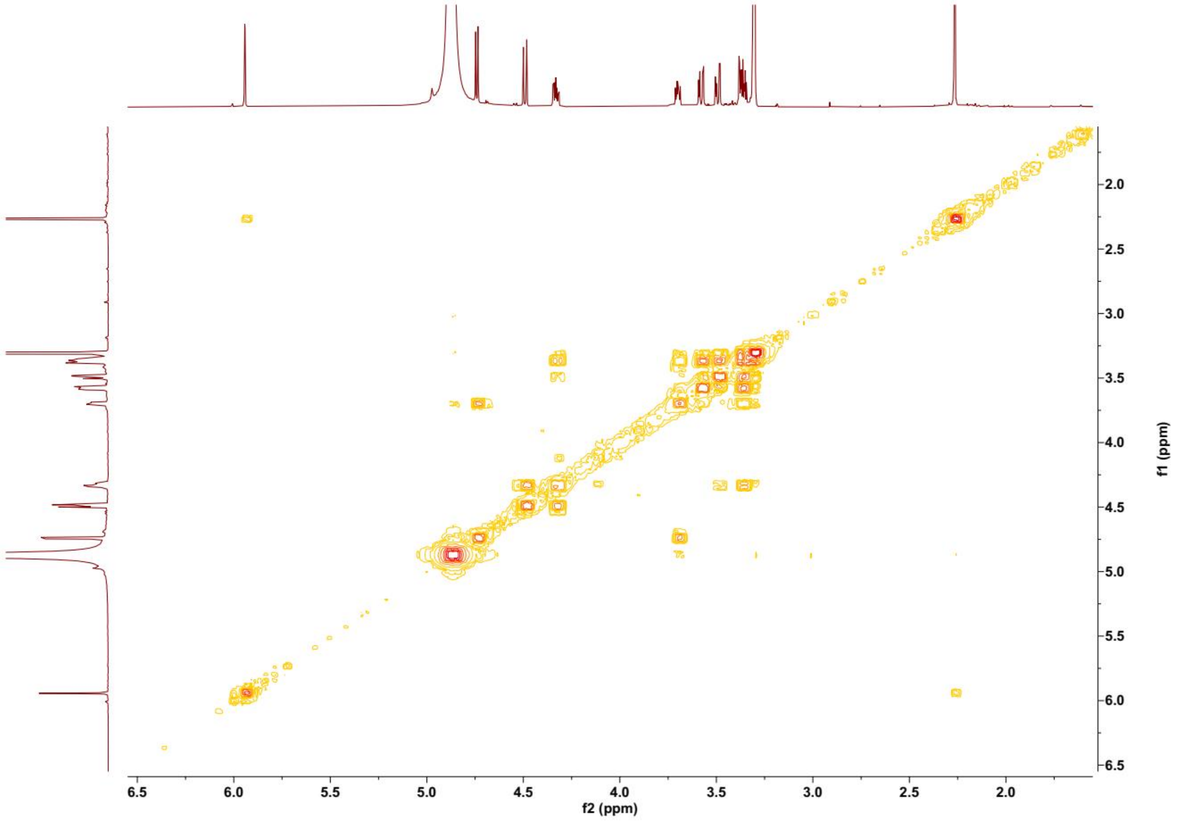

Figure S39. ^1H-^1H COSY spectrum of **5** in methanol-d_4

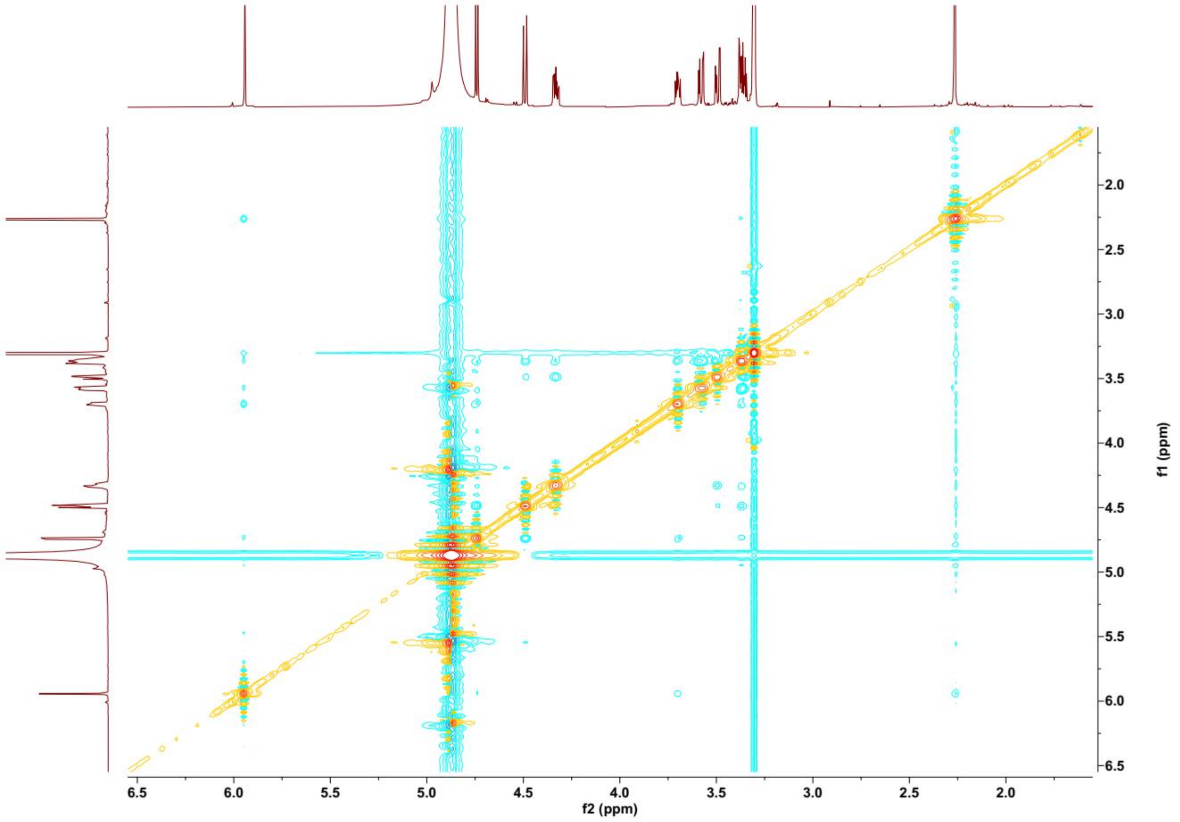

Figure S40. NOESY spectrum of **5** in methanol-*d*4

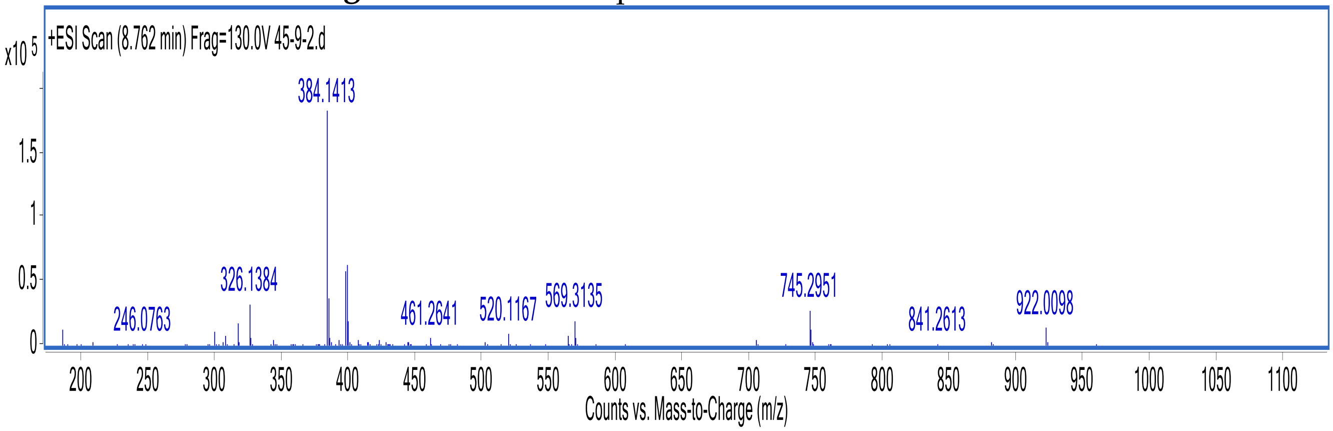

Figure S41. HRESIMS spectrum of **5**
